# Multiple loci linked to inversions are associated with eye size variation in species of the *Drosophila virilis* phylad

**DOI:** 10.1038/s41598-020-69719-z

**Published:** 2020-07-30

**Authors:** Micael Reis, Gordon Wiegleb, Julien Claude, Rodrigo Lata, Britta Horchler, Ngoc-Thuy Ha, Christian Reimer, Cristina P. Vieira, Jorge Vieira, Nico Posnien

**Affiliations:** 1 Department of Developmental Biology, Göttingen Center for Molecular Biosciences (GZMB), University of Goettingen, Justus-von-Liebig-Weg 11, 37077 Göttingen, Germany; 2 International Max Planck Research School for Genome Science, Am Fassberg 11, 37077 Göttingen, Germany; 3 Institut Des Sciences de l’Evolution de Montpellier, CNRS/UM2/IRD, 2 Place Eugène Bataillon, cc64, 34095 Montpellier Cedex 5, France; 4 Instituto de Investigação e Inovação em Saúde, Universidade do Porto, Porto, Portugal; 5 Instituto de Biologia Molecular e Celular (IBMC), Universidade do Porto, Porto, Portugal; 6 Animal Breeding and Genetics Group, Department of Animal Sciences, University of Goettingen, Albrecht-Thaer-Weg 3, 37075 Göttingen, Germany; 7 Center for Integrated Breeding Research, University of Goettingen, Albrecht-Thaer-Weg 3, 37075 Göttingen, Germany

**Keywords:** Genome-wide association studies, Structural variation, Evolutionary genetics

## Abstract

The size and shape of organs is tightly controlled to achieve optimal function. Natural morphological variations often represent functional adaptations to an ever-changing environment. For instance, variation in head morphology is pervasive in insects and the underlying molecular basis is starting to be revealed in the *Drosophila* genus for species of the *melanogaster* group. However, it remains unclear whether similar diversifications are governed by similar or different molecular mechanisms over longer timescales. To address this issue, we used species of the *virilis* phylad because they have been diverging from *D. melanogaster* for at least 40 million years. Our comprehensive morphological survey revealed remarkable differences in eye size and head shape among these species with *D. novamexicana* having the smallest eyes and southern *D. americana* populations having the largest eyes. We show that the genetic architecture underlying eye size variation is complex with multiple associated genetic variants located on most chromosomes. Our genome wide association study (GWAS) strongly suggests that some of the putative causative variants are associated with the presence of inversions. Indeed, northern populations of *D. americana* share derived inversions with *D. novamexicana* and they show smaller eyes compared to southern ones*.* Intriguingly, we observed a significant enrichment of genes involved in eye development on the *4th* chromosome after intersecting chromosomal regions associated with phenotypic differences with those showing high differentiation among *D. americana* populations. We propose that variants associated with chromosomal inversions contribute to both intra- and interspecific variation in eye size among species of the *virilis* phylad.

## Introduction

One of the most important goals of biological research is to understand the mechanisms underlying morphological diversification. The molecular basis of simple morphological traits, such as pelvic reduction in sticklebacks^1^, presence or absence of trichomes in *Drosophila*^2^, and pigmentation variation in flies^3,4^ and mice^5^, have been determined and are usually caused by a small number of large effect loci. However, the molecular basis of variation in complex traits remains largely elusive.

The insect head represents a great model to study complex trait evolution, since it harbors major sensory organs, which facilitate fundamental processes like feeding and reproduction. Natural variation in insect head size and shape is pervasive in insects and it is often driven by a functional trade-off between visual and olfactory sensory investment^6–12^, suggesting that it is likely caused by functional adaptations to an ever-changing environment. Externally, this trade-off is often observed by extensive head shape variation if compound eye size increases at the expense of the cuticle between the eyes (i.e. interstitial head cuticle)^6,13–15^. The compound eyes are the most noticeable sensory structures in the insect head and differences in eye size have been reported between species, as well as between populations of the same species across the *Drosophila* genus^6,7,13–17^. Interestingly, eye size can vary due to variation in facet size or due to changes in ommatidia number^13,14,17,18^, suggesting that different functional needs influence final eye size.

Quantitative genetics approaches have revealed multiple loci associated with variation in eye size between *D. simulans* and *D. mauritiana* supporting the complex genetic architecture of this trait^17^. Similar observations were made for intraspecific variation in *D. melanogaster*^7,19^ and *D. simulans*^14^. However, a single mutation affecting the regulation of the *eyeless/Pax6* gene was shown to explain up to 50% of variation in eye size between two *D. melanogaster* strains^7^. Although, the genetic architecture underlying eye size variation is starting to be revealed for species of the *melanogaster* group, it remains unclear whether similar independent morphological diversifications identified in *Drosophila*^6,15^ share the same molecular basis over longer timescales.

Chromosomal inversions are an interesting genetic variant because suppression of recombination is thought to maintain linkage of favorable alleles which are protected from immigrant alleles carrying variants which decrease fitness^20,21^. Therefore, chromosomal inversions can act as super genes influencing a myriad of phenotypes that can have a large adaptive value. The impact of chromosomal inversions on many life-history and physiological traits is well established and is often associated with local adaptation^22–25^. Additionally, chromosomal inversions are associated with differences in rather simple morphological traits. For instance, natural variation in chromosomal inversions affect wing, thorax and head phenotypes in *D. buzzatii*^26,27^ and wing size and shape in *D. mediopunctata*^28^ and in *D. melanogaster*^29^. Chromosomal inversions are commonly associated with population structure and hinder the distinction between correlated and causative variants^30^. Therefore, the impact of inversions on the diversity of complex morphological traits remains largely elusive.

Species of the *virilis* phylad of *Drosophila* are diverging from *D. melanogaster* for at least 40 million years^31,32^ and they have been extensively used in comparative genomics studies of important ecological traits, such as body color^4,33^, cold resistance^34^, life span^35^ and developmental time^36^. *D. virilis* is a cosmopolitan species of Asian origin while *D. americana* and *D. novamexicana* are endemic to the USA^37^ and constitute the *americana* complex. *D. americana* shows a wide geographical distribution along the eastern part of the USA while *D. novamexicana* has a smaller distribution in the south-central part of the USA^38^. *D. novamexicana* originated from a peripheral population of the last common ancestor of *D. americana*/*D. novamexicana* less than 1 million years ago and it has been evolving in complete isolation since there is no evidence for introgression between these two species^39^. Several chromosomal inversions segregate in *D. americana* populations showing latitudinal and longitudinal gradients^37,40^. Some of these inversions create highly differentiated genomic regions between northern and southern *D. americana* populations and they are shared between northern *D. americana* populations and *D. novamexicana*^41^. Since species of the *virilis* phylad, and in particular the *americana* complex, have multiple well characterized chromosomal inversions and extensive phenotypic variability, these species are a prime model to link variation in phenotypes to the presence of chromosomal inversions and simultaneously understand whether natural variation in organ morphology is due to the same molecular basis in divergent *Drosophila* lineages.

In this work we provide a comprehensive morphological and genetic characterization of eye size variation among species of the *virilis* phylad. We show that eye size differences are most pronounced between *D. novamexicana* and a southern strain of *D. americana*. Applying quantitative genetics approaches we establish that eye size differences are caused by multiple genes located in multiple chromosomes. Additionally, we found an association between the presence of chromosomal inversions and eye size. A thorough integration of population genetics, GWAS and phylogenic datasets revealed a significant enrichment for eye developmental genes among genes located on the *4th* chromosome (Muller B). We argue that some of these variants can explain both intra- and interspecific variation in eye size.

## Results

### Head shape and eye size are remarkably variable in species of the *virilis* phylad

**Figure 1:**
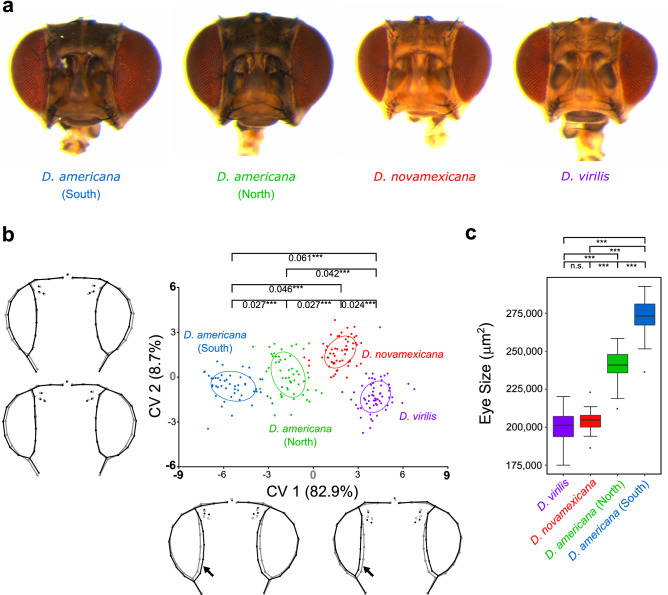
Eye size and head shape are remarkably variable among species of the *virilis* phylad. (**a**) Heads of representatives of each strain/population showing shape and corrected eye size closest to the median (**b**) Head shape variation among species/populations after Canonical Variate Analysis (CVA) of the Procrustes coordinates obtained after correcting for error and transposing the orthogonal matrix containing the first 12 principal components explaining 90.8% of the total variation in head shape (see “Materials and methods” for details). Each point refers to one individual and equal frequency ellipses are given with probability of 0.5. Procrustes distances (sum of the differences between comparable landmarks of mean shape conformations) between groups are presented above the ellipses (***P < 0.0001 after a permutation test with 10,000 iterations). The black wireframes depict changes in shape along the two main canonical variates (CV1 and CV2) per unit of within-group variation [Mahalanobis distances: ± 9 (CV1) and ± 6 (CV2)] relative to the grey wireframes (CV = 0 for each axis). The arrows depict the most pronounced changes in the ventral region of the head. The amount of variation explained by each CV is shown in brackets. (**c**) Eye size variation (after accounting for variation in body size) among species/populations (Kruskal–Wallis test, followed by post-hoc Dunn’s test and Holm correction for multiple testing: *P < 0.05, ***P < 0.001, n.s.P > 0.05).

To evaluate the extent of variation in overall head shape in the *virilis* phylad, we performed a geometric morphometrics analysis to quantify shape differences in females of two strains of *D. virilis*, *D. novamexicana*, a northern and a southern population of *D. americana*, respectively (Fig. 1a). The mean shapes were significantly different for all possible pairwise comparisons among species/populations (Fig. 1b). We found that bigger eyes were associated with reduced interstitial cuticle and this effect was more pronounced in the ventral part of the head (Fig. 1b).

To confirm the observed variation in eye size, we measured eye area in females of each species/population. We observed that southern *D. americana* strains had the largest eyes while *D. virilis* and *D. novamexicana* had the smallest (Fig. 1c). Differences in eye size reached 36.2% when southern *D. americana* strains were compared to *D. virilis* and 13.7% between *D. americana* populations (Supplementary File S1 online). Therefore, differences in head shape are accompanied by eye size (after accounting for variation in body size) variation and this association was further confirmed by the significant correlation between the former trait and Canonical variate 1 (CV1) (Pearson’s r = − 0.925, P < 2.2e−16). Overall, these results show that eye size and head shape differ remarkably between species of the *virilis* phylad and among *D. americana* populations.

### Variation in head shape and eye size is associated with chromosomal inversions in strains of the *americana* complex

Chromosomal inversions are pervasive in the *virilis* phylad^37,40^. Therefore, the observed variation in eye size and head shape provides an excellent model to test whether inversions are associated with differences in complex morphological traits. We developed new molecular markers for each chromosomal inversion and genotyped all analyzed strains (see “Materials and methods”). Our results were largely compatible with previous observations (Supplementary File S2 online^37,40^). Inversions *Xa* (Muller A) and *2a* (Muller E) were exclusive of *D. virilis*, while inversion *Xb* (Muller A) was present in all *D. novamexicana* and *D. americana* strains. Inversions *2b* (Muller E) and *5b* (Muller C) were exclusive of *D. novamexicana*, while inversion *5a* (Muller C) was exclusive of *D. americana*. Inversions *Xc* (Muller A) and *4a* (Muller B) were present in *D. novamexicana* and *D. americana* (O43, O53). For inversion *5a* (Muller C) we found evidence for heterozygosity in *D. americana* (O43) (*5a*/*5*). Surprisingly, we could not find evidence for the presence of inversion *5b* (Muller C) in northern *D. americana* strains, which was previously described to be fixed in northern populations^40^.

**Figure 2:**
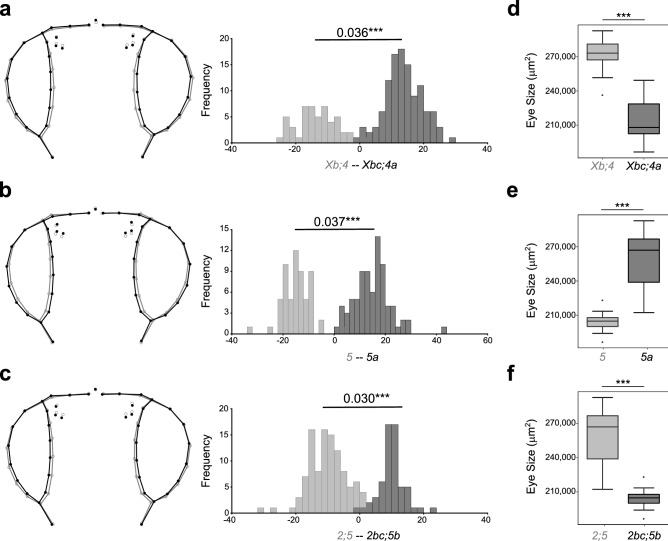
Variation in eye size and head shape is strongly associated with the presence of chromosomal inversions among species of the *americana* complex. (**a–c**) Head shape variation among species/populations with (dark grey) or without (light grey) chromosomal inversions *Xc*,*4a* (**a**), *5a* (**b**), and *2bc*,*5b* (**c**). The histograms depict the frequency (*y*-axis) of the discriminant scores (*x*-axis) obtained after Discriminant function analysis based on the Procrustes coordinates obtained from the first 12 principal components explaining 90.8% of the total variation. Procrustes distances between groups are provided along with the histograms (***P < 0.0001 after a permutation test with 1,000 iterations). The wireframes depict changes in the mean shape (grey—without inversions; black—with inversions). (**d**–**f**) Eye size variation (after accounting for variation in body size) between species/populations with (dark grey) or without (light grey) chromosomal inversions *Xc*,*4a* (**d**), *5a* (**e**), and *2bc*,*5b* (**f**) (Wilcoxon rank-sum test and Holm correction for multiple testing: ***P < 2.2e−6).

Since most of the inversions, except *Xa* and *2a*, are derived in the lineage leading to *D. americana* and *D. novamexicana*^37,41^, we excluded *D. virilis*, to address the impact of inversions on head shape and eye size (after accounting for variation in body size) in the *americana* complex. We found significant associations between the presence of inversions and head shape variation, mostly affecting the ratio between eye size and the head cuticle (Fig. 2a–c). Accordingly, we also found significant associations between the presence of inversions and eye size among strains (*Xbc,4a* v. *Xb,4* (Muller A, B) (W = 5,489, P < 2.2e−16); *2bc,5b* v. *2,5* (Muller E, C) (W = 6,113, P < 2.2e−16) (Fig. 2d–f). The presence of inversions *Xc,4a* (*D. novamexicana* and northern *D. americana*) and *2b,5b* (*D. novamexicana*) resulted in a 19.1% and 20.3% reduction in eye size, respectively. Inversion *5a* (*D. americana*) led to a significant increase of 26.8% (*5a* v. *5* (W = 7, P < 2.2e−16). These results suggest that at least part of the causative variants underlying variation in eye size and head shape must be located in chromosomal inversions.

### F1 hybrids between *D. americana* and *D. novamexicana* exhibit intermediate eye size and head shape

The strains showing the largest differences in eye size (after accounting for body size) were *D. americana* (SF12) and *D. novamexicana* (15010-1031.00) (Supplementary File S1 online). Additionally, with respect to chromosomal inversions they showed the most divergent karyotypes, supporting the association between inversions and eye size. Therefore, we selected those two strains to characterize head shape and eye size variation and dominance relationship more comprehensively.

**Figure 3:**
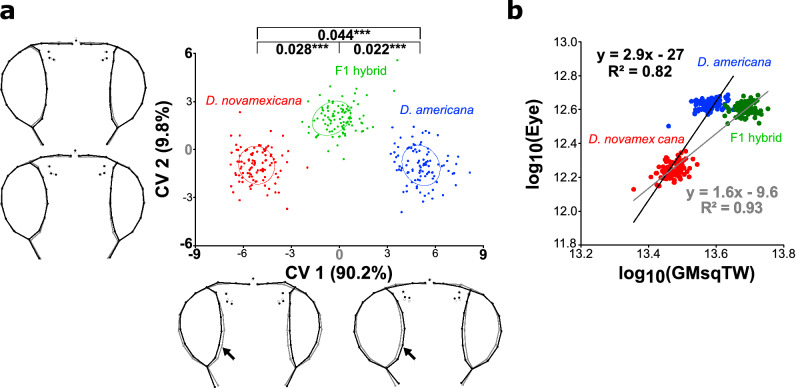
Eye size and head shape are significantly different between *D. americana* and *D. novamexicana* and their interspecific hybrids show intermediate phenotypes. (**a**) Head shape variation between parental strains (*D. novamexicana*, N = 99, *D. americana*, N = 100) and their interspecific hybrids (N = 100). Canonical Variate Analysis (CVA) was applied to the Procrustes distances obtained after correcting for error and transposing the orthogonal matrix containing the first nine principal components explaining 90.5% of the total variation in head shape (see “Materials and methods” for details). Each point refers to one individual and equal frequency ellipses are given with probability of 0.5. Procrustes distances (sum of the differences between comparable landmarks of mean shape conformations) between groups are presented above the ellipses (***P < 0.0001 after a permutation test with 10,000 iterations) The black wireframes depict changes in shape along the two main canonical variates (CV1 and CV2) per unit of within-group variation [Mahalanobis distances: ± 9 (CV1) and ± 6 (CV2)] relative to the grey wireframes (CV = 0 for each axis). The amount of variation explained by each CV is shown between brackets. The arrows depict the most pronounced changes in the ventral region of the head. (**b**) Scaling relationships between females of *D. novamexicana* (15010-1031.00) (N = 99) and *D. americana* (SF12) (N = 98), as well as between females of *D. novamexicana* and F1 interspecific hybrids (progeny of crosses between *D. novamexicana* males and *D. americana* females; N = 98) for eye area relatively to the GMsqTW. The slopes of the equations represent the allometric coefficients between *D. novamexicana* and F1 hybrids (grey) as well as between *D. novamexicana* and *D. americana* (black).

We performed a geometric morphometrics analysis to evaluate differences in head shape between both parental strains and their F1 hybrids. We found that head shapes were significantly different for all comparisons (Fig. 3a). The main differences between the species and their hybrid was explained by CV1 with the hybrid showing an intermediate head shape. CV1 captured an expansion of the eye that was accompanied by a contraction of the interstitial cuticle. This effect was more pronounced in the ventral region for *D. americana* (Fig. 3a).

Next, we compared eye areas (frontal view) of F1 hybrids to the parental strains and found for all comparisons statistically significant differences (Kruskal–Wallis test followed by Dunn’s post-hoc test, P < 0.001; Supplementary File S1 online), with *D. americana* females having 46.4% larger eyes than *D. novamexicana* females. The eyes of hybrids were slightly, but significantly smaller (2.4%) than *D. americana* female eyes (Supplementary File S1 online). To determine whether differences in eye size were caused by variation in ommatidia number, size, or both, we measured the area and counted ommatidia of eyes (lateral view, see “Materials and methods” for details). Although we cannot exclude regional differences in ommatidia size between species, ommatidia counting strongly suggests that eye size differences were exclusively caused by variation in ommatidia number (Supplementary Fig. S1 online).

To test whether body size influenced the observed eye size differences, we measured wing areas as well as tibiae lengths in both parental strains and in hybrids. All comparisons between the two strains and their interspecific hybrids were statistically significant (Kruskal–Wallis test followed by Dunn’s post-hoc test, P < 0.001; Supplementary File S1 online). Interestingly, while eye area was slightly, but significantly smaller in hybrids than in *D. americana* , the other organs were larger in F1 hybrids (Fig. 3b; Supplementary File S1 online) leading to significantly larger allometric coefficients between *D. novamexicana* and *D. americana* when compared to *D. novamexicana* and F1 hybrids (B = 1.30, P < 0.001; Fig. 3b). This result suggests that part of the difference in eye size between the F1 hybrids and *D. novamexicana* may be caused by pronounced changes in total body size. In summary, our results show that eye size and head shape are significantly different between *D. novamexicana* and *D. americana* and intermediate in hybrids.

### Relative eye size and head shape is affected by variation on multiple chromosomes

**Figure 4:**
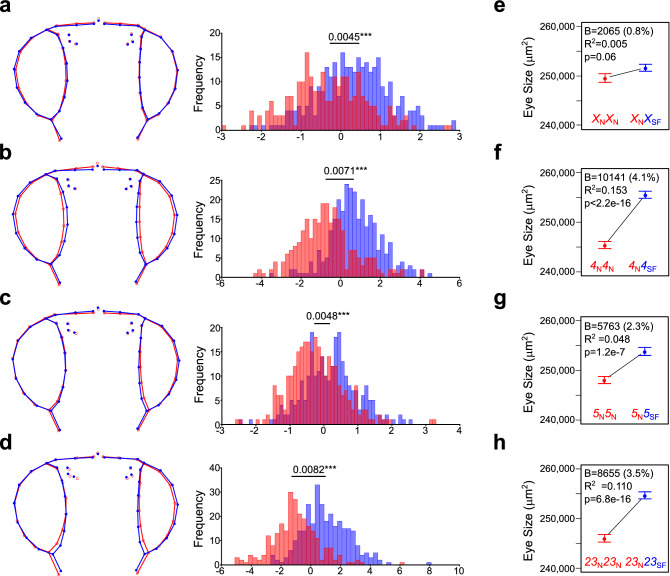
Variation in relative eye size and head non-allometric shape is mainly explained by the *2nd* and *3rd* fused chromosomes (Muller E and D) as well as by the *4th* chromosome (Muller B). (**a**–**d**) Variation in mean head shape among the female progeny of the backcross between F1 hybrid females and *D. novamexicana* males. The histograms depict the frequency (*y*-axis) of the discriminant scores (*x*-axis) obtained after Discriminant function analysis of the Procrustes coordinates obtained from the first 19 principal components (90.8% of the total variation). Procrustes distances between groups are provided along with the histograms (***P < 0.0001 after a permutation test with 1,000 iterations). The wireframes depict changes in the mean shape multiplied by a factor of 5 [homozygous *D. novamexicana* (red) or heterozygous *D. novamexicana*/*D. americana* (blue)]. (**e**–**h**) Distributions of relative eye size (plots of means ± SEM) for females, progeny of the backcross, which were homozygous for a given *D. novamexicana* chromosome (red) or heterozygous *D. novamexicana*/*D. americana* (blue) for the respective chromosome. Information about the magnitude of change in eye size, the significance values as well as the percentage of variation explained obtained using linear regression models is shown inside the graphs.

To reveal genetic variants associated with head shape and eye size differences between *D. americana* and *D. novamexicana*, we performed a backcross study (see “Materials and methods” for details). Most chromosomes were associated with the size of multiple adult organs simultaneously with a pronounced effect of the *5th* chromosome (Muller C) (Supplementary Fig. S2 online), suggesting that variants in general factors affecting overall body size are segregating in these crosses. Therefore, we evaluated the effect of individual chromosomes on the non-allometric component of shape (Fig. 4a–d; for the effect of each chromosome on the allometric shape see Supplementary Fig. S3 online). We found significant associations between every chromosome and head shape variation, with smaller effects of the *X* or *5th* chromosomes (Muller A or C) compared to the *2nd*, *3rd*, and *4th* chromosomes (Muller E, D, and B) (Fig. 4a–d). The differences in mean shape caused by the *D. americana* fused *2nd* and *3rd* chromosomes (Muller E and D) and the *4th* chromosome (Muller B) were compatible with a trade-off between the eyes and the interstitial cuticle (Fig. 4b, d). This effect was only observed in the ventral region of the head for *2nd* and *3rd* chromosomes (Muller E and D) (Fig. 4d) and the presence of the *4th* chromosome (Muller B) additionally caused an expansion of the eye area in the lateral part of the head (Fig. 4b). Hence, genes located on all chromosomes contribute to head shape variation between *D. americana* and *D. novamexicana*.

Next, we assessed the effect of each chromosome on eye size. Since variants affecting overall body size segregated in our crosses, we determined which chromosomes affect eye size exclusively. To this end, we adopted a very conservative approach to account for variation in overall body size (see “Materials and methods” for details). After accounting for differences in total body size, we observed that the *X* chromosome (Muller A) had no significant effect, while all other chromosomes showed a strong association with the relative eye size (Fig. 4e–h). The main effects were caused by the presence of the *4th* chromosome (Muller B) (Fig. 4f) and the fused *2nd* and *3rd* chromosomes (Muller E and D) (Fig. 4h), which explained 15.3% and 11.0% of the variation and resulted in an increase of 4.1% and 3.5% in relative eye size, respectively. We also found a slight contribution of the *5*^*th*^ chromosome (Muller C) (Fig. 4g) which explained 4.8% of the variation and led to an increase of 2.3% in relative eye size.

We did not find evidence for epistasis between the chromosomes showing significant associations with relative eye size (RelE ~ Ch23 × Ch4; RelE ~ Ch23 × Ch 5; RelE ~ Ch4 × Ch5; and RelE ~ Ch23 x Ch4 × Ch5, P > 0.05 for all interactions), suggesting that the contribution of the different chromosomes was additive. This result is further supported by the observation that the presence of the fused *2nd* and *3rd* chromosomes (Muller E and D), the *4th* chromosome (Muller B) or the *5th* chromosome (Muller C) by themselves contributed very little to an increase in relative eye size (Supplementary Fig. S4 online). Indeed, the genotypic class showing the highest values of relative eye size was the one heterozygous for all chromosomes except the *X* chromosome (Muller A) (11.8% bigger than the class having *D. novamexicana* chromosomes only, Supplementary File S1 online). We conclude that genes located on the *D. americana 2nd*, *3rd*, *4th* and *5th* chromosomes (Muller E, D, B, and C) when present simultaneously on a *D. novamexicana* background contribute additively to the highest increase in relative eye size.

To increase the mapping resolution and to reveal SNPs associated with relative eye size we performed a Genome-Wide Association Study (GWAS) using pools of individuals after 17 generations of recombination between hybrids (see “Materials and methods” for details). The results obtained were highly compatible with our backcross study. We found clear regions with major differentiation between extreme quartiles on the *2nd* and *3rd* chromosomes (Muller E and D), as well as on the *4th* chromosome (Muller B) and the *5th* chromosome (Muller C) (Supplementary Fig. S5 online). Additionally, we confirmed that the chromosomal inversions segregating in our crosses largely suppress recombination even after 17 generations. Further analysis of intermediate quartiles showed that the frequencies of the reference variants increased for different chromosomes between adjacent quartiles (Supplementary Fig. S5 online). Increased relative eye size is, thus, caused by combinations of different chromosomes and it is largest when the frequencies of *D. americana* variants are highest across the genome. Overall, these results represent compelling evidence for the role of multiple genes located in different chromosomes in relative eye size determination.

### Variants located in genes involved in eye development can explain both intra- and interspecific variation in relative eye size

**Figure 5:**
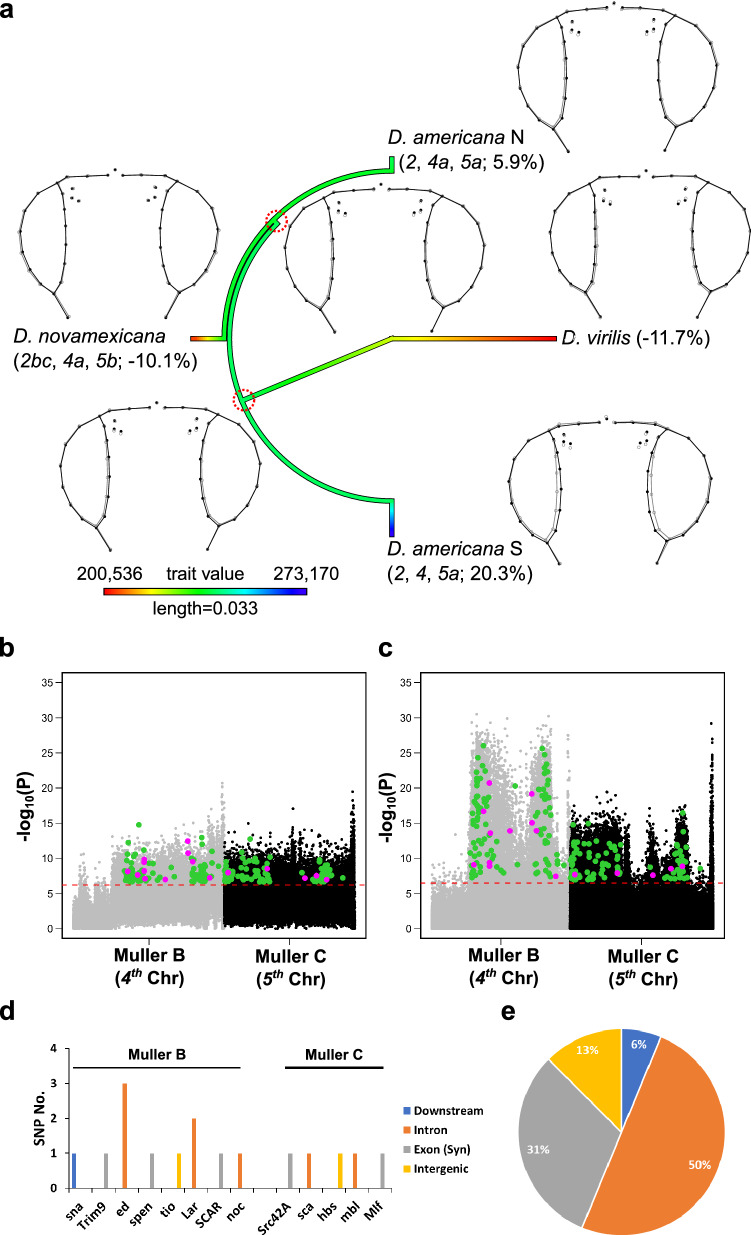
Phylogenetic and population genetics approaches revealed loci linked with inversions that can explain variation in eye size in species of the *virilis* phylad. (**a**) Ancestral reconstruction of the ancestral eye size (after accounting for variation in body size) of species of the *virilis* phylad. The karyotypes, the wireframes (black—mean head shape of each species/population, grey—mean head shape of the estimated ancestral), and the percentage of variation in eye size compared to the grand mean are shown for each species/population. (**b**, **c)** Manhattan plots of the data obtained between Q4 and Q1 (F18 pool-seq) and between southern and northern *D. americana* populations, respectively for Muller B and C. The green dots depict the SNPs showing significant differences in frequencies in both data sets after Bonferroni correction; the purple dots depict the subset of significant SNPs located inside or nearby candidate genes for eye development. (**d**) Regions of candidate genes involved in eye development where the SNPs depicted in purple in (**b**, **c**) were located. (**e**) Frequency of the SNPs located in different gene regions.

To narrow down the high number of potential variants (SNPs) obtained from our GWAS approach, we integrated population genetics data from *D. americana*. Under a simple additive model, the sum of the effects of the different chromosomes lead to the overall effect observed. In southern *D. americana* populations (e.g. SF12), this leads to bigger eyes while in *D. novamexicana* this leads to smaller eyes (Figs. 5a and 1b). The highly differentiated regions between northern and southern *D. americana* populations^41^ should be at least partly shared between northern populations and *D. novamexicana*, because they share inversions *Xc* (Muller A), *4a* (Muller B) and *5b* (Muller C) (Fig. 5a, Supplementary Fig. S6 online, but see previous results). In contrast, chromosomes not showing differentiation (*2nd* and *3rd*, Muller E and D) are shared between northern and southern populations (Supplementary Fig. S6 online) and when combined with *Xc*, *4a* and *5b* chromosomes resulted in an intermediate eye size in northern *D. americana* populations (Fig. 5a and Fig. 1b). However, the *2nd* and *3rd* chromosomes show extensive differentiation between *D. novamexicana* and *D. americana* alleles after 17 generations of recombination (Supplementary Fig. S5 online), due to the presence of inversions *2b*, *2c* and *3a*, which are fixed in *D. novamexicana*^37,40^ and contributed to a smaller eye size (Figs. 5a and 1b). Therefore, we raised the hypothesis that regions showing high differentiation due to the presence of inversions will contain the variants associated with differences in eye size in the *americana* complex. Following this assumption, we intersected the SNPs showing significantly higher frequency of the reference variant [*D. americana* (SF12)] in Q4 compared to Q1 with those showing the reference variants at higher frequencies in southern *D. americana* populations^41^ (see Supplementary Fig. S7 online and “Materials and methods”for details). This analysis resulted in 41,840 SNPs (Supplementary Fig. S7 online).

To further narrow down the number of meaningful SNPs we integrated phylogenetic data. We reconstructed the ancestral state of size traits to understand how these phenotypes evolved in the *virilis* group of species. We observed that the ancestral eye size was intermediate (Fig. 5a) and this result was supported by the ancestral reconstruction of size phenotypes of 59 *Drosophila* species reported in^6^ (Supplementary Fig. S8 online). Given the high levels of phenotypic and nucleotide variation characteristic of *D. americana*^35^, it is likely that the intermediate ancestral eye size represented the mean of a quantitative trait including smaller and bigger eyes. Thus, we assumed that the ancestral population of the *virilis* phylad was highly polymorphic for eye size, and both bigger and smaller eye size were selected for in specific lineages from a pool of standing genetic variation (Fig. 5a). Under this hypothesis, the variants responsible for increased eye size should be fixed in the *D. americana* (SF12) reference genome and at higher frequencies in southern populations, while the alternative variants should be fixed between *D. novamexicana* and *D. virilis*. Therefore, we kept only SNPs that showed the alternative variant common between *D. novamexicana* and *D. virilis* from the list of SNPs showing significant differences in frequency between extreme quartiles of the GWAS between *D. americana* and *D. novamexicana*. For this final list of SNPs, we annotated them to genes using the information available for *D. virilis*.

We obtained a total of 6,670 SNPs within or close to 3,006 unique genes. However, only 2,627 unique genes of *D. virilis* have recognizable orthologs in *D. melanogaster* (1,254; 534; 366 and 473 on the *2nd*, *3rd*, *4th* and *5th* chromosomes (Muller E, D, B and C), respectively; Supplementary File S3 online). Next, we identified the orthologs of these 2,627 *D. virilis* genes in *D. melanogaster* (2,701 genes due to some ambiguities, Supplementary File S3 online) and intersected them with the 397 genes associated with eye development (see “Materials and methods” for details). We obtained 446 SNPs located within or close to 126 unique genes (59, 22, 21 and 24 on the *2nd*, *3rd*, *4th* and *5th* chromosomes (Muller E, D, B, and C), respectively Supplementary File S3 online). These results showed that one fifth of the total number of genes estimated to be present in *D. melanogaster* (2,701/13,767) had SNPs with significant frequency differences in the GWAS and the same variant in *D. novamexicana* and *D. virilis*, but a different variant in *D. americana* (SF12). Almost one third of the total number of candidate genes for eye development (126/397) were present among those genes. Thus, in our dataset we observed a significant over-representation of eye developmental genes (126 out of 2,701 vs. 397 out of 13,701, Chi-square statistics with Yates correction = 37.25, p < 1.00e−5) that may explain variation in relative eye size among species of the *virilis* phylad.

We also postulated that the combination of different variants shared either with *D. novamexicana* or with southern *D. americana* populations leads to an intermediate phenotype in northern *D. americana* populations (Fig. 5a). Thus, the overlap between the chromosomal regions associated with phenotypic differences with those showing high differentiation among *D. americana* populations^41^ may explain both inter- and intraspecific variation in eye size. The intersection of both datasets resulted in a total of 119 SNPs in 102 genes on the *4th* chromosome (Muller B) and 101 SNPs in 102 genes on the *5th* chromosomes (Muller C) (Supplementary File S3 online). After intersecting these 204 genes with the 397 candidates for eye development (see “Materials and methods” for details), we obtained 11 SNPs in 8 out of 102 genes on the *4th* chromosome, and 5 SNPs on 5 out of 102 genes in the *5th* (Fig. 5b–c). There was a significant over-representation of genes involved in eye development on the *4th* chromosome (8 out of 102 vs. 397 out of 13,701; Chi-square statistics with Yates correction = 7.13, P = 7.6e−3), but this was not the case for the *5th* (5 out of 102 vs. 397 out of 13,701; Chi-square statistics with Yates correction = 0.84, P = 0.36) (Fig. 5d). Most of the identified SNPs were in non-coding regions (69%), suggesting that regulatory sequences and thus associated gene expression may be predominantly affected (Fig. 5e). As expected, most of the SNPs that can explain both intra- and interspecific variation in relative eye size were located on the *4th* and *5th* chromosomes (Muller B and C). However, we also observed two SNPs in two genes on the *2nd* chromosome (Muller E) and four SNPs in four genes on the *3rd* chromosome (Muller D). One of the SNPs on the *3*^*rd*^ chromosome was a synonymous mutation located in one candidate gene for eye development) (Supplementary File S3 online). In summary, we revealed a significant enrichment in genes involved in eye development on the *4th* chromosome carrying variants strongly associated with relative eye size variation between species of the *virilis* phylad and within *D. americana*.

## Discussion

We provide the most comprehensive morphological and molecular characterization of head shape and eye size variation in species outside the *melanogaster* group. We observed remarkable differences in these two traits among species of the *virilis* phylad. Our shape analysis revealed that increased eye size was accompanied by a contraction of the interstitial head cuticle. A similar trade-off has been observed in other *Drosophila* species^6,7,13–15,17^ and it may be associated with different investment in visual or olfactory sensory perception^6,7^. Indeed, it has been shown that a northern *D. americana* strain is more “visual” because it has significantly bigger eyes compared to the antennae, while *D. virilis* is a more “olfactory” species with smaller eyes and bigger antennae^6^. Following this rational, *D. novamexicana* may be a more “olfactory” species compared to the southern *D. americana* strain studied in detail here.

While differential investment in visual or olfactory sensory information is a common phenomenon in animals^6–12^, it remains to be established whether the genetic underpinnings are the same or not. Since the compound eyes (i.e. vision) and the antennae (i.e. olfaction) originate from the same imaginal disc during larval development^42^, the pervasive variation in head shape and eye size in *Drosophila* is an excellent model to test this. Variation in eye size between *D. melanogaster* strains is highly associated with one SNP in the Cut transcription factor binding site in the *eyeless/Pax6* regulatory region^7^. However, *eyeless/pax6* and *cut* are located on Muller F and Muller A, respectively. Since these chromosomes are not associated with differences in eye size in our study, those two genes cannot explain the natural variation in eye size observed among species of the *virilis* phylad. Also, data obtained for *D. mauritiana* and *D. simulans* showed that differences in eye size are due to variation in facet size^13^, while eye size differences between *D. novamexicana* and *D. americana* are mostly caused by differences in ommatidia number. Since facet size and ommatidia number are specified by different developmental processes^43,44^, it is likely that different developmental mechanisms underly natural variation in eye size. Indeed, the most significant QTL explaining eye size variation in *D. mauritiana* and *D. simulans* mapped to the *X* chromosome^17^. In contrast, our data showed that genetic variants affecting exclusively eye size were located in all chromosomes, but not in the *X* and *6th* chromosomes (Muller A and F). Therefore, our comparative morphological and mapping data strongly suggest an independent evolution of eye size in different lineages. This observation is supported by similar data obtained for *D. melanogaster* and *D. simulans* of the *melanogaster* group^14^.

The imaginal disc that gives rise to the *Drosophila* head is a modular structure that contributes cells to almost all organs of the head^42^. Since we observed an additive effect of *D. americana* chromosomes in a *D. novamexicana* background on eye size, it is conceivable that each chromosome or chromosomal region might influence different developmental processes and different organ anlagen within the imaginal disc. This hypothesis is supported by our observation that the *D. americana 5th* chromosome (Muller C) had a major impact on the size of all organs in our study, while the *2nd*, *3rd* and *4th* chromosomes (Muller E, D and B) were associated with variation in eye size after accounting for body size. Please note that we cannot rule out the presence of variants located on the *5th* chromosome (Muller C) affecting exclusively eye size that were masked by the conservative approach for body size correction used in this work. Our shape analysis further revealed that only the *4th* chromosome (Muller B) was associated with variation in lateral eye regions, supporting a modular impact of different chromosomes on overall head shape variation. For species of the *melanogaster* group it has also been shown that the evolution of eye size and the size of the interstitial cuticle is uncoupled^14,17^. In contrast, the SNP in the *eyeless/Pax6* locus associated with intraspecific variation in *D. melanogaster* influences the early subdivision of the imaginal disc into the retinal and the antennal part of the imaginal disc^7^. Therefore, this SNP may affect eye size and head cuticle/antennal size simultaneously. Although more comparative developmental analyses are necessary, the picture emerges that the modular nature of the imaginal disc with its different interconnected developmental programs may facilitate the independent evolution of head shape because it provides multiple targets for evolutionary changes.

In our survey we observed the most pronounced differences in eye size between *D. novamexicana* showing the smallest eyes and southern *D. americana* strains showing the largest eyes. Interestingly, compatible with studies using species of the *melanogaster* group^7,13–15,19^, we also identified intraspecific differences among *D. americana* populations. There are multiple chromosomal inversions segregating in *D. americana* populations and some of them are shared with *D. novamexicana*^37,40^. These inversions largely affected the patterns of differentiation along chromosomes among *D. americana* natural populations^41^. Although the genomic structure caused by the presence of inversions hampers the identification of causative variants, it has been proposed that they may keep together favorable combinations of alleles^20,21^. Interestingly, we observed a clear association between the presence of shared inversions and eye size. Additionally, we found a number of genes involved in eye development among those genes containing SNPs that could explain both intra- and interspecific differences in eye size on the *4th* (Muller B) and *5th* (Muller C) chromosomes. Among those genes we found several candidates that interact with key signaling pathways involved in eye development, such as Notch (*hbs*^45^, *sca*^46^, *noc*^47^, *spen*^48^, *Ed*^49^) and Epidermal growth factor receptor (EGFR) signaling (*Src42A*^50^,* spen*^48^,* Ed*^51^). Intriguingly, we found three genes in our candidate list which have been directly implicated in growth control during eye and head development. The gene *tiptop* (*tio*) codes for a highly conserved Zinc finger transcription factor that can induce ectopic eyes upon ectopic in the antennal anlagen and it regulates cell proliferation during eye development^52^. Similarly, loss of function of the Zinc finger transcription factor No ocelli (Noc) results in overgrowth of head cuticle tissue^47^, suggesting that it is involved in regulating eye vs. head cuticle growth. And the Myelodysplasia/myeloid leukemia factor (Mlf) has been implicated in controlling the transition from cell proliferation to differentiation in the developing eye^53^, a crucial process in final eye size control. These genes represent prime candidates for future functional validation tests.

Since the inversions shared by *D. americana* and *D. novamexicana* show latitudinal and longitudinal gradients, it is likely that they carry the targets of selection associated with local adaptation in natural populations. For instance, chromosomal inversions were found to be associated with life-history and physiological traits, such as life span, fecundity and cold tolerance, likely involved in adaptation^22–24^ as well as with morphological traits, such as body and wing size and shape^26–29^. Additionally, a previous study found that the fixed variant explaining pigmentation differences between *D. novamexicana* and *D. americana* was polymorphic in *D. americana* and explained the least pronounced variation in pigmentation observed along a longitudinal transect in *D. americana* populations^4^. Therefore, it is conceivable that interspecific differences affecting exclusively eye size between *D. novamexicana* and *D. americana* can also explain intraspecific differences in this trait among *D. americana* natural populations. The longitudinal gradient for pigmentation in *D. americana* populations was further confirmed^33^, and solar radiation and the diurnal temperature range has been shown to be the best predictors of this gradient^54^. Interestingly, *D. americana* populations showing darker pigmentation were more often found in geographical regions with lower sun radiation and mean diurnal temperature ranges than lighter populations^54^. According to these geographical parameters, we observed here that flies showing bigger eye size, likely more sensitive to light than flies with smaller eyes, came from regions with low sun radiation and possibly with less light. Therefore, eye size variation in the *americana* complex may be associated with local adaptation as well.

In conclusion, natural variation in head morphology is common in *Drosophila* and has a strong genetic component. We provide for the first time a comprehensive morphological comparison of eye size and head shape between *D. novamexicana* and *D. americana* and revealed a complex underlying genetic architecture. Our data strongly suggests that the presence of inversions in these two species contributed to nucleotide diversity patterns that may affect the regulation and function of multiple genes during head and eye development and thus facilitating natural variation in this complex morphological trait.

## Materials and methods

### Fly strains

The following isofemale fly strains were used in this work: *D. virilis* (15010-1051.47, Hangzhou, China; 15010-1051.49, Chaco, Argentina), *D. novamexicana* (15010-1031.00, Grand Junction, Colorado, USA; 15010-1031.04, Moab, Utah, USA) and *D. americana* (O43, O53, SF12 and SF15). The *D. virilis* and *D. novamexicana* strains were obtained from the Tucson stock center in 1995 and were kept in the laboratory since then. *D. americana* strains were established with single inseminated females collected from the wild in different locations of the USA (Omaha, Nebraska (O), 2008 and Saint Francisville (SF), Louisiana, 2010)^34,35,55^. All strains were kept at 25 °C under 12 h/12 h light/dark cycles.

### Dissection and phenotyping

To study size variation, we dissected heads, wings and tibiae of 20–30 females between 4 and 7 days after eclosion for each strain. To avoid crowding effects on adult organ size, we controlled for density by transferring 30 first instar larvae into single vials containing standard food. The heads were mounted facing upwards on a slide with sticky tape, while the three legs (one of each pair) and wings were randomly dissected from the left or right side and mounted on a slide with Hoyer’s medium. Pictures were taken using a stereomicroscope Leica M205 FA with a magnification of 50 × for wings and 60 × for the other structures. We also took a picture of a ruler to be able to convert the measurements from pixels to µm or µm^2^. The resulting JPG files were saved with a resolution of 2,560 × 1920 pixels, and we used ImageJ^56^ to measure eye areas as well as tibiae lengths and wing areas (Supplementary Fig. S9 online, Supplementary File S4 online). We calculated the geometric mean of squared tibiae (GMsqT) as a proxy for tibiae size. The geometric mean of squared tibiae and wing area (GMsqTW) was used to estimate overall body size. We then used the residuals of the linear regression between eye size and GMsqTW to account for differences in overall body size between the strains. Since the measurements were not normally distributed (Shapiro–Wilk, P < 0.05), we used Kruskal–Wallis test followed by Dunn’s post-hoc test with Holm correction to determine which comparisons were significantly different between strains.

### Geometric morphometrics

Frontal head images of every strain were used to generate tps files in which all images were randomized with tpsUtil (version 1.60;^57^). These tps files were used to place 43 landmarks and semilandmarks (see Supplementary Fig. S9 online) using tpsDig2 (version 2.18;^57^). A sliders file that contains information about the semilandmarks was generated with the “Make sliders file” function in tpsUtil (version 1.60;^57^). Using tpsRelw (version 1.57, 64 bit;^57^) the semilandmarks were slid along a curve using an option to minimize the bending energy required for a deformation of the consensus to the selected specimen (Slide method = Chord min BE) allowing up to three iterations during the superimposition process (Slide max iters = 3). The slid landmarks were treated as fixed landmarks and were superimposed using Procrustes fit as implemented in MorphoJ (version 1.06d;^58^). Since we used 2D pictures of 3D structures, after a principal component analysis (PCA) we observed that artificial pitch (up/down rotation) and yaw (left/right rotation) were partly associated with shape variation along principal component (PC) 1 and PC3 axes, respectively (Supplementary Fig. S10 online). Additionally, we observed that part of the within-strain variation captured pitch and yaw. To dissociate and remove the error from the true components of shape, we used a two-step approach: First, we calculated the residuals of the within-strain pooled-regression between Procrustes coordinates and PC1. Second, we used the new coordinates to repeat the above-mentioned procedure to remove the effect of yaw (new PC2). To avoid inflation of the number of the variables when compared to the number of samples in the statistical analysis, we performed a PCA on the final Procrustes coordinates to determine and keep the number of PCs explaining about 90% of the total shape variation. The reduced PC dataset was rotated back into Procrustes coordinates by transposing the orthogonal matrix. Differences in shape among species/strains were evaluated using a T-square parametric test followed by 10,000 permutations (leave-one-out cross-validation) as implemented in the “Canonical Variate Analysis” option in MorphoJ. The resulting main canonical variates are those explaining most of the variation in shape among groups after accounting for within-group variation and are expressed as Mahalanobis distances^59^. The MorphoJ output also provides the differences in shape and significance values for every pairwise comparison expressed either as Mahalanobis distances or as Procrustes distances (square root of the sum of squared distances between the coordinates of comparable landmarks of two superimposed shape configurations^60^). The error correction and wireframes generation were performed with MorphoJ while the PC removal and rotation to the original coordinates was done using a custom R script. All raw pictures of wings, legs and heads as well as files generated for the geometric morphometrics analyses are available online (https://doi.org/10.25625/PKDVFT).

### Impact of chromosomal inversions on eye size

To test whether the presence of inversions was associated with differences in eye size (after accounting for body size) and head shape, we screened *D. americana*, *D. novamexicana* and *D. virilis* strains for the presence/absence of eight different inversions known to be fixed or polymorphic within the *virilis* phylad of *Drosophila*^37,40^ and for which the breakpoint locations have been identified (*Xa*, *Xb*, *Xc* (Muller A), *2a*, *2b* (Muller E), *4a* (Muller B), *5a* and *5b* (Muller C),^41,61,62^). Primers were developed for one breakpoint and its corresponding ancestral state for each of the eight inversions (Supplementary File S2 online) based on the *D. virilis*^63^, *D. americana* (H5, W11^35^ and SF12^41^), and *D. novamexicana* (15010-1031.00^41^) genome sequences to avoid polymorphism at the primer binding sites. Genomic DNA was extracted from pools of 20 females for each strain using a standard phenol:chloroform protocol, and the concentration was normalized based on concentration measurements using Nanodrop prior to PCR amplification (Supplementary File S2 online). The amplification products were visualized on a UV transilluminator after electrophoresis using TAE buffer in 2% agarose gels stained with a 1:10 dilution of SERVA stain. Associations between the presence of inversions and eye size (after accounting for body size as described above) were tested using Wilcoxon rank-sum test followed by Holm correction for multiple testing. Associations between mean head shape variation and the presence of inversions were tested using a parametric T-square test on the group mean shapes followed by 1,000 permutations (leave-one-out cross-validation) as implemented in the “Discriminant Function Analysis” option in MorphoJ.

### Parental strains selection and hybrid phenotyping

*D. americana* (SF12) and *D. novamexicana* (15010-1031.00) strains were selected as representatives of both species, because they had the largest differences in eye size (see Results), they show the most divergent karyotypes regarding chromosomal inversions and their genomes are available^41^. We established several crosses with 10 males and 10 females for each of both parental strains, and 10 *D. novamexicana* males and 10 *D. americana* females to obtain F1 hybrids. Since, *D. americana* females and males take at least four to six days to reach full maturity^64^, we transferred the flies into new vials after seven days. The flies were then allowed to lay eggs for 24 h only, to avoid crowding effects on adult size before dissection. Newly eclosed flies were sexed and collected into new vials and were kept under the same conditions described above. Previous studies have shown that *Drosophila* males are smaller than females, but the differences between strains are preserved when comparing males or females^13,14^. Therefore, we used females only to avoid hemizygosity for the *X* chromosome. We dissected 100 females of each parental strain, as well as 100 F1 females between 4 and 7 days after eclosion, as described above. Pictures were taken using a stereomicroscope Nikon ZMS 1,500 H with a magnification of 40 × for wings and 50 × for the other structures. We also took a picture of a ruler to be able to convert the measurements to µm or µm^2^. The resulting JPG files were saved with a resolution of 1,600 × 1,200 pixels. All pictures were treated using ImageJ^56^ as mentioned before. After phenotyping, six individuals were excluded from the analysis because they showed highly damaged wings (Supplementary File S4 online). We used Kruskal–Wallis test followed by Dunn’s post-hoc test with Holm correction to determine which comparisons were significantly different between strains and hybrids. The scaling relationships between parental strains, as well as between *D. novamexicana* and F1 hybrids were evaluated using the regression of log-transformed eye area (non-corrected for body size) on log-transformed GMsqTW. The slope of the resulting curves is the allometric coefficient^65,66^. We tested for the significance of differences in allometric coefficients using a linear model with interaction terms.

The geometric morphometric analysis of head shape including removal of artificial pitch and yaw was done as described above (“Geometric morphometrics” section).

For ommatidia counting, the heads of 10 females for each parental strain and hybrids were dissected 4–7 days after eclosion and cut in half longitudinally with a razor blade. One of the eyes of every individual was mounted on sticky tape facing upwards. Serial stack pictures (N = 25) were taken using a microscope Zeiss Axioplan 2 with external light sources from the sides and 160X magnification to capture the reflection of every ommatidia (Supplementary Fig. S1 online). We also took a picture of a ruler to be able to convert the measurements from pixels to µm or µm^2^. Images were saved with 1,360 × 1,036 resolution. Stack projection with maximum intensity was achieved using ImageJ^56^. The area of the eye was outlined and measured. The images were transformed into 8-bit (gray scale) and the area outside the selected region was cleared. Next, we used the Fast_Morphology.jar plug-in with the following settings: morphological filters, white Tophat–octagon–radius = 2. The images were inverted and the ommatidia numbers were estimated using the ITCN_1_6.jar plug-in with the following settings: width = 7px; Minimum distance = 10, threshold = 2.0 and detect dark peaks. To estimate the average ommatidia size, the eye area was divided by the number of ommatidia. All raw pictures of wings, legs and heads as well as files generated for the geometric morphometrics analyses are available online (https://doi.org/10.25625/J9MV6V).

### Genotype–phenotype association study using a backcross approach

To determine the effect of the major chromosomes on size variation, we established backcrosses between F1 females (progeny of crosses between *D. novamexicana* males and *D. americana* females) and *D. novamexicana* males. A total of 570 females were dissected and phenotyped for eye, face and wing areas, as well as for tibiae lengths as described above. After phenotyping, 11 females were excluded from the analysis because they showed highly damaged wings (Supplementary File S4 online). The remaining 559 females were genotyped using the molecular markers A6 (Muller A, *X* chromosome), B3 (Muller B, *4th* chromosome), C3 and C5 (Muller C, *5th* chromosome), D7 (Muller D, *3rd* chromosome) and E7 (Muller E, *2nd* chromosome) (see^36^ and Supplementary File S5 online for more details). PCR reactions were done using Phire Plant Direct PCR kit (Thermo Scientific) and gDNA from wings according to the manufacturer’s instructions. We found some unspecific amplification with molecular markers A6 and B3. Based on the recently published *D. americana* (SF12) and *D. americana* (15010-1031.00) genomes^41^, we were able to slightly modify these primers to account for polymorphisms and improve PCR amplification (Supplementary File S5 online). No recombinants were found between molecular markers C3 and C5 on the *5th* chromosome (Muller C). The *2nd* and *3rd* chromosomes (Muller E and D, respectively) are fused in *D. americana*. These are, thus, transmitted as a single chromosome and we found only four recombinants between the molecular markers D7 and E7 (Supplementary File S55 online). We excluded the four recombinants and also three individuals that showed the amplification product of *D. americana* only for marker A6 or E7 (Supplementary File S5 online). This cleaned data set of 552 females was used to evaluate the effect of each chromosome in eye and wing areas, as well as in GMsqT with the Wilcoxon-rank test followed by Holm correction for multiple testing. Since we observed a strong effect of the *5th* chromosome (Muller C) on the size of all analyzed organs (see Results), we decided to use the residuals of the multiple linear regression of eye size on tibiae and wing sizes to account for body size variation. With this conservative approach we removed all the variation in eye size that could be explained by variation in the other structures. We have summed the grand mean of eye size to the residuals to get relative eye size. Since relative eye size was normally distributed (Shapiro–Wilk test, P > 0.05), we used linear models with each chromosome as fixed effect to test for significant associations and to estimate the amount of variation explained. We have further included interaction terms between different chromosomes to evaluate epistasis.

The geometric morphometric analysis of head shape was mainly done as described above. In this analysis, pitch and yaw were partly associated with variation along PC1 and PC2, respectively. The error was removed prior to the analysis using the method described before. The impact of the different chromosomes on mean shape variation was evaluated using the “Discriminant Function Analysis” option in MorphoJ. We further used the residuals of the regression of the Procrustes coordinates on centroid size to estimate the impact of the different chromosomes on the non-allometric component of shape. These analyses were also conducted in MorphoJ. All raw pictures of wings, legs and heads of the 570 backcross females as well as files generated for the geometric morphometrics analyses are available online (https://doi.org/10.25625/HRYOBC).

### Genome-wide association study using a pool-seq approach

#### Crosses, sample preparation and sequencing

To identify single nucleotide polymorphisms (SNPs) associated with relative eye size, we performed a genotype–phenotype association study using F18 individuals resulting from brother sister mating for 17 generations starting from crosses between *D. novamexicana* (15010-1031.00) males and *D. americana* (SF12) females. We phenotyped a total of 157 females, which represented the entire F18 female progeny of two independent vials, for eye and wing areas, as well as tibiae lengths of one of each pair of legs. Wings and legs were randomly dissected from left or right sides. Missing values for individuals ID = 125 and 150 showing highly damaged wings were estimated based on the equation of the multiple linear regression between wing area and tibiae length. The residuals of the multiple regression between eye area and both tibiae lengths and wing areas were used to remove the variation in eye area that could be explained by variation in total body size. The grand mean was summed to the residuals and these new values were sorted in ascending order to divide the females into four quartiles [Q1 (n = 40; 238,913 ± 7,479µm^2^); Q2 (n = 39; 252,838 ± 2,352µm^2^), Q3 (n = 38; 263,652 ± 3,777µm^2^) and Q4 (n = 40; 278,609 ± 9,736µm^2^] (Supplementary File S4 online). All raw pictures of the F18 female wings, legs and heads are available online (https://doi.org/10.25625/NHEKGJ). gDNA for pooled carcasses was extracted for each quartile using a standard phenol:chloroform procedure. DNA quantity and integrity were checked by agarose gel electrophoresis. The good quality samples were used to prepare gDNA libraries for all four pooled samples with TruSeq Nano DNA Library Prep from Illumina (Catalog#FC-121-9010DOC). The libraries were further used for paired-end sequencing with HiSeq2000 (Illumina) at the Transcriptome Analysis Laboratory (TAL) in Göttingen.

#### Quality checks and analysis

After sequencing using HiSeq2000, a total of 88,783,338; 75,693,540; 82,724,476 and 108,310,437 paired-end reads with 100 bp were obtained for Q1, Q2, Q3 and Q4, respectively. The reads are available at BioProject PRJNA641990. The quality of the reads was assessed with FASTQC v0.11.1 (https://www.bioinformatics.babraham.ac.uk/projects/fastqc/). There was no need to trim or mask positions, since all positions had quality above 20. As mapping reference the *D. americana* (SF12) genome was used after it was reordered based on the hypothetical chromosomes of the ancestral state between *D. virilis*, *D. americana* and *D. novamexicana*^41^. Read mapping, ambiguously mapped reads and optical duplicates removal, as well as SNP calling and depth of coverage determination were done as described in^41^. The overall alignment rates using Bowtie2 v2.2.5^67^ with default settings for Q1, Q2, Q3, and Q4 were 77%, 79%, 79% and 81%, respectively. The distributions of coverage obtained with GATK DepthOfCoverage v3.4.46^68^ were close to normal and the average values for Q1, Q2, Q3, and Q4 were 84X, 73X, 81X and 107X, respectively. For all quartiles, more than 94% of the sites showed coverage values above 20X. To avoid bias in frequency estimations, we used a coverage interval which included 68.2% of the total amount of sites around the mean for each quartile ([62-101X], [54-89X], [60-97X] and [84-127X] for Q1, Q2, Q3 and Q4, respectively). The data was treated and analyzed as described in^41^. Briefly, we used the frequencies values for SNPs identified using both Bowtie2 v2.2.5^67^ and BWA v0.7.12^69^ to determine which ones showed significant differences between the quartiles using Fisher exact test followed by Bonferroni correction.

### Ancestral reconstruction of phenotypic traits

We used the phylogeny of 59 *Drosophila* species obtained by^6^ to reconstruct the ancestral state for body size, eye surface, as well as the ratio between eye and head width using a Maximum Likelihood method as implemented in the function fastAnc in the R package Phytools (v. 0.4.98, https://www.phytools.org/eqg2015/asr.html^70^). The details about the strains and the phenotypes can be found in^6^.

We also used Phytools to reconstruct the ancestral state of different traits for the species of the *virilis* phylad. The traits considered were the following: GMsqTW as a proxy for body size, eye area, the ratio between eye and head area, and eye area after accounting for body size (residuals of the linear regression between eye area and GMsqTW plus the grand mean of eye area). To obtain phylogenies representative of the *virilis* phylad, we started by downloading all the *D. virilis* coding sequences (CDS) available at FlyBase (ftp://ftp.flybase.net/genomes/Drosophila_virilis/current/fasta/). We further used SEDA^71^ to retrieve only those CDS of genes located on scaffolds anchored to chromosomes (Muller E: scaffolds 12,822; 13,047; 12,855 and 12,954; Muller B: scaffolds 13,246; 12,963 and 12,723 and Muller C: scaffolds 12,823; 10,324; 12,875 and 13,324). When more than one isoform was available for the same gene only the longest one was retrieved. Next, we used Splign-Compart (as implemented in BDBM^72^, and the *D. virilis* CDS obtained above as references to annotate CDS in *D. americana* (H5 and W11^35^; SF12, Northern, Central and Southern populations^41^), as well as in *D. novamexicana* (15010-1031.00) contigs^41^. To obtain the CDS for the *D. americana* populations, we used Coral 1.4^73^ with default parameters to reconstruct the gene sequences showing the major frequent variant at polymorphic sites in the pool-seq reads of each population^41^, prior to contig assembly using Abyss 2.0^74^ with K = 25 and default parameters. For each genome, we used SEDA to filter the datasets for complete CDS (those with annotated start and stop codons) and obtain one file per gene with the orthologous CDS. Sequences were aligned using Clustal Omega^75^ and concatenated, resulting in alignments with 372,546 bp (379 genes), 335,931 bp (379 genes) and 298,233 (334 genes) for the *2nd*, *4th* and *5th* chromosomes (Muller E, B, and C), respectively. FASTA files were converted to NEXUS format using ALTER^76^. The phylogenies were obtained with MrBayes^77^ using the GTR model of sequence evolution allowing for among-site rate variation and a proportion of invariable sites. Third codon positions were allowed to have a different gamma distribution shape parameter than those for first and second codon positions. Two independent runs of 1,000,000 generations with four chains each (one cold and three heated chains) were used. Trees were sampled every 100th generation and the first 2,500 samples were discarded (burn-in) (Supplementary Fig. S11 online). The Docker images used for running the above software applications are available at the pegi3s Bioinformatics Docker Images Project (https://pegi3s.github.io/dockerfiles/). Since we have no phenotypic data for *D. americana* H5, W11, and the Central population, their branches were manually removed from the phylogenies. The distances between nodes were accounted for to re-estimate the new branch lengths when applicable (Supplementary Fig. S11 online). We kept the Northern population branch as a phylogenetic proxy for O43 and O53 phenotypes and we decided to remove the Southern population branch because we have genomic data for SF12. The phylogenies were rooted by the *D. virilis* branch prior to ancestral state reconstruction of the size phenotypes using Phytools as described above and ancestral state reconstruction of shape using squared-changed Parsimony^78^ as implemented in the option “Map Onto Phylogeny” in MorphoJ.

### Intersection with previous results and SNP annotation

To identify variants associated with relative eye size and linked with chromosomal inversions, we intersected the SNPs obtained for the F18 pool-seq described above with those obtained in a previously published pool-seq of *D. americana* populations^41^ (Supplementary Fig. S7 online). We started by re-mapping the raw reads obtained for the genome sequencing of *D. americana* (SF12) and *D. novamexicana* (15010-1031.00) against the reference *D. americana* (SF12) genome. Read quality was assessed with FastQC v0.11.1 (https://www.bioinformatics.babraham.ac.uk/projects/fastqc/), and every position showing a quality score under 20 was masked using fastq_masker implemented in the FASTX Tool kit v.0.0.13 (https://hannonlab.cshl.edu/fastx_toolkit/index.html). Read mapping, alignment filtering and SNP calling was done as described above. The mean coverage and standard deviation (s.d.) were determined for both samples and all SNPs showing lower or higher frequency than mean ± 3 s.d. were considered either as errors or originating from highly repetitive regions and were discarded. Next, every SNP showing coverage higher than zero for the reference variant in *D. novamexicana* (15010-1031.00) and the alternative one in *D. americana* (SF12) was excluded. These were variants that might be shared between the two strains, and they would cause biased frequencies. Then, these tables were intersected with those obtained for Q1 and Q4 of the F18 pool-seq described above. Fisher exact test followed by Bonferroni correction was used to determine which SNPs show significant frequency differences between Q1 and Q4. We kept those SNPs which show the reference allele at higher frequencies for Q4. Since, *D. virilis* and *D. novamexicana* show small eye size (see Results), we considered that the variants causing this phenotype would be fixed in those species. The alternative variant should be fixed in *D. americana* (SF12) and at higher frequencies in Q4 and southern populations.

To annotate the SNPs to genes, we started by aligning the *D. americana* (SF12) reference genome to the *D. virilis* genome using Mauve v2.4.0^79^. The raw SNPs coordinates were extracted and were converted according to the position and orientation of the different *D. virilis* scaffolds to match the coordinates provided on the gtf file (ftp://ftp.flybase.net/genomes/Drosophila_virilis/dvir_r1.07_FB2018_05/gtf/). The gtf file was converted to genePred format using gtfToGenePred tools (https://hgdownload.soe.ucsc.edu/admin/exe/linux.x86_64/). A file containing all the transcripts of *D. virilis* was generated using the genePred file and the FASTA file (dvir-all-predicted-r1.07.fasta.gz) with all predicted annotations available for *D. virilis* (ftp://ftp.flybase.net/genomes/Drosophila_virilis/dvir_r1.07_FB2018_05/fasta/) using the script retrieve_seq_from_fasta.pl provided in ANNOVAR v. (Mon, 16 Apr 2018), (https://www.openbioinformatics.org/annovar/annovar_download_form.php^80^. The SNPs were then annotated using the script ./table_annovar.pl provided in ANNOVAR.

To determine whether some of the identified SNPs were located in genes associated with eye development, we intersected the list of genes with those described as being involved in “eye development” (GO: 001654; https://flybase.org, FB2018_03). We observed that the *D. virilis* orthologs of three genes (*PQBP1*, *CkIIalpha*, and *hyd*) are located on scaffold 12,958. Since this scaffold is not anchored to any Muller element in *D. virilis*, we excluded those genes from the analysis. We also decided to include the manually curated genes *ewg* and *pnr* which have been reported to be involved in eye development (FlyOde; https://flyode.boun.edu.tr/^81^, but are not present in the GO term: “eye development”.

To identify those regions that can explain both intra- and interspecific variation in relative eye size, we have further filtered the SNPs which showed significant frequency differences between northern and southern *D. americana* populations after Bonferroni correction. Gene enrichment was tested using Chi-square test with Yates correction. To obtain an estimation of the total number of genes in *D. melanogaster*, we obtained the list of genes in the Geneontology Panther Classification system (https://pantherdb.org/). This list was used to retrieve the chromosomal location in Flybase (https://flybase.org/batchdownload). Only those genes located on Muller A, B, C, D, E and F were considered (Supplementary File S3 online).

## Supplementary information

Supplementary Information 1.

Supplementary Information 2.

Supplementary Information 3.

Supplementary Information 4.

Supplementary Information 5.

Supplementary Information 6.

## Data Availability

All raw pictures are available online (https://doi.org/10.25625/NHEKGJ; https://doi.org/10.25625/HRYOBC; https://doi.org/10.25625/PKDVFT; https://doi.org/10.25625/J9MV6V), and raw measurements in Supplementary File S4 online. Unless stated differently, all statistical analyses presented in this work were done using R^82^ and the R package Rcmdr^83–85^. The plots were prepared using ggplot2^86^ and Microsoft Office. All custom scripts, analysis pipelines (https://doi.org/10.25625/VZKP3Y) and MorphoJ project files (https://doi.org/10.25625/H6ALY4) are available online. Raw reads used for the F18 pool-seq analysis are available via BioProject PRJNA641990.
