# Supplementary material for: Multiple loci linked to inversions are associated with eye size variation in species of the Drosophila virilis phylad
Source: Sci Rep. 2020 Jul 30;10:12832. doi: 10.1038/s41598-020-69719-z (PMC7393161; doi:10.1038/s41598-020-69719-z)
Supplement: Supplementary file 1 — Supplementary Information 1. [file 41598_2020_69719_MOESM1_ESM.pdf]

Supplementary Information

Multiple loci linked to inversions are associated with eye size variation in species of the *virilis* phylad

Reis, Micael[1]; Wiegleb, Gordon[1,2]; Claude, Julien[3]; Lata, Rodrigo[4,5]; Horchler, Britta[1]; Ha, Ngoc-Thuy[6,7]; Reimer, Christian[6,7]; Vieira, Cristina P.[4,5]; Vieira, Jorge[4,5]; Posnien, Nico[1,*]

[1]University of Goettingen, Department of Developmental Biology, Göttingen Center for Molecular Biosciences (GZMB), Justus-von-Liebig-Weg 11, 37077 Göttingen, Germany
[2]International Max Planck Research School for Genome Science, Am Fassberg 11, 37077 Göttingen, Germany
[3]Institut des Sciences de l'Evolution de Montpellier, CNRS/UM2/IRD, 2 Place Eugène Bataillon, cc64, 34095 Montpellier Cedex 5, France
[4]Instituto de Investigação e Inovação em Saúde, Universidade do Porto, Portugal
[5]Instituto de Biologia Molecular e Celular (IBMC), Universidade do Porto, Portugal
[6]University of Goettingen, Animal Breeding and Genetics Group, Department of Animal Sciences, Albrecht-Thaer-Weg 3, 37075 Göttingen, Germany
[7]University of Goettingen, Center for Integrated Breeding Research, Albrecht-Thaer-Weg 3, 37075 Göttingen, Germany

*corresponding author: nposnie@gwdg.de

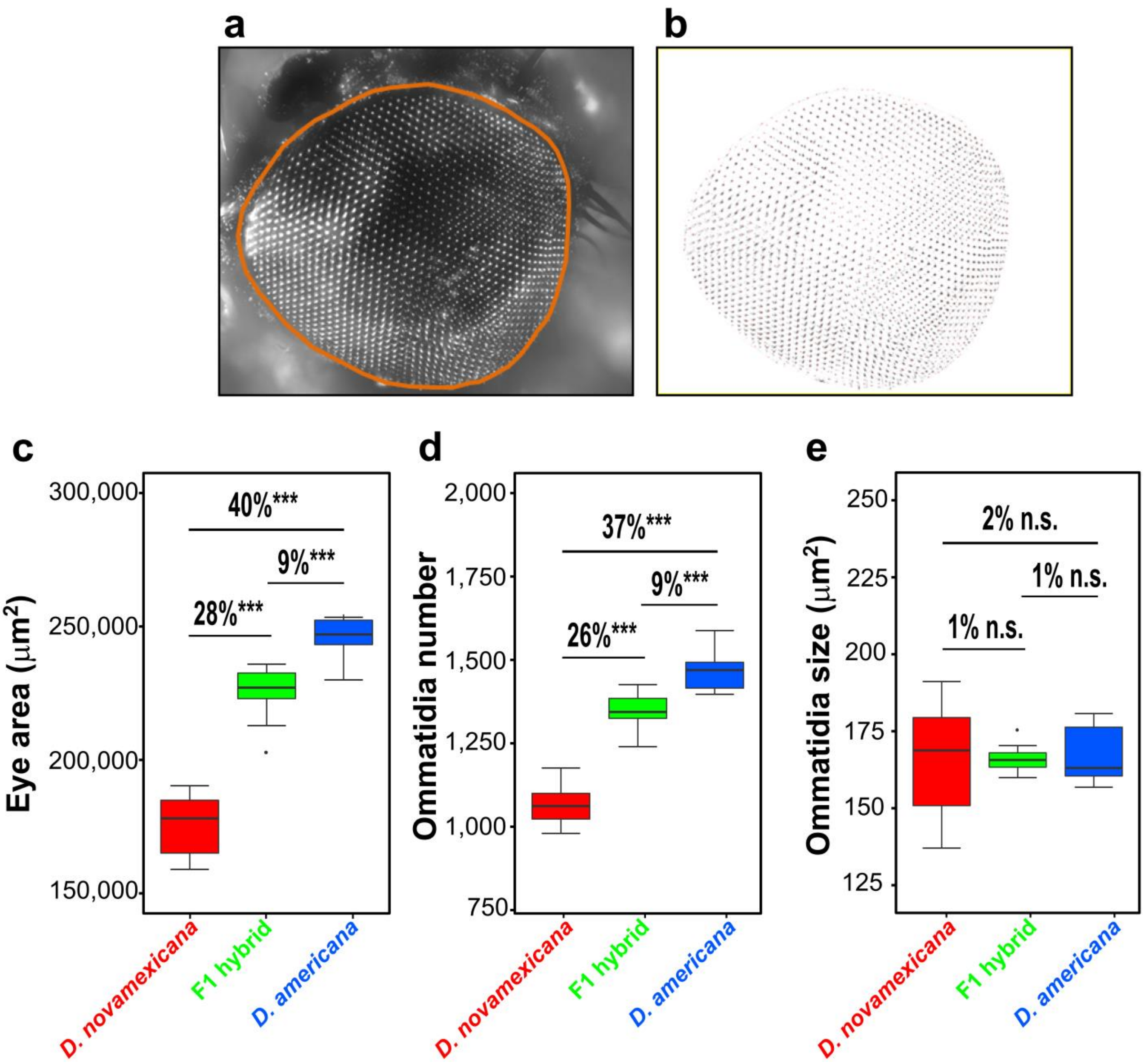

Supplementary Fig. S1. Differences in eye size, ommatidia number, and ommatidia size between parental strains and their interspecific hybrid. a. The measured eye area is depicted in orange. **b.** Transformed picture used to count ommatidia. **c.** Variation in eye area among parents (10 females of each) and hybrids (10 females). **d.** Variation in ommatidia number for the same individuals. **e.** Average ommatidia size obtained as the ratio between eye area and ommatidia number.

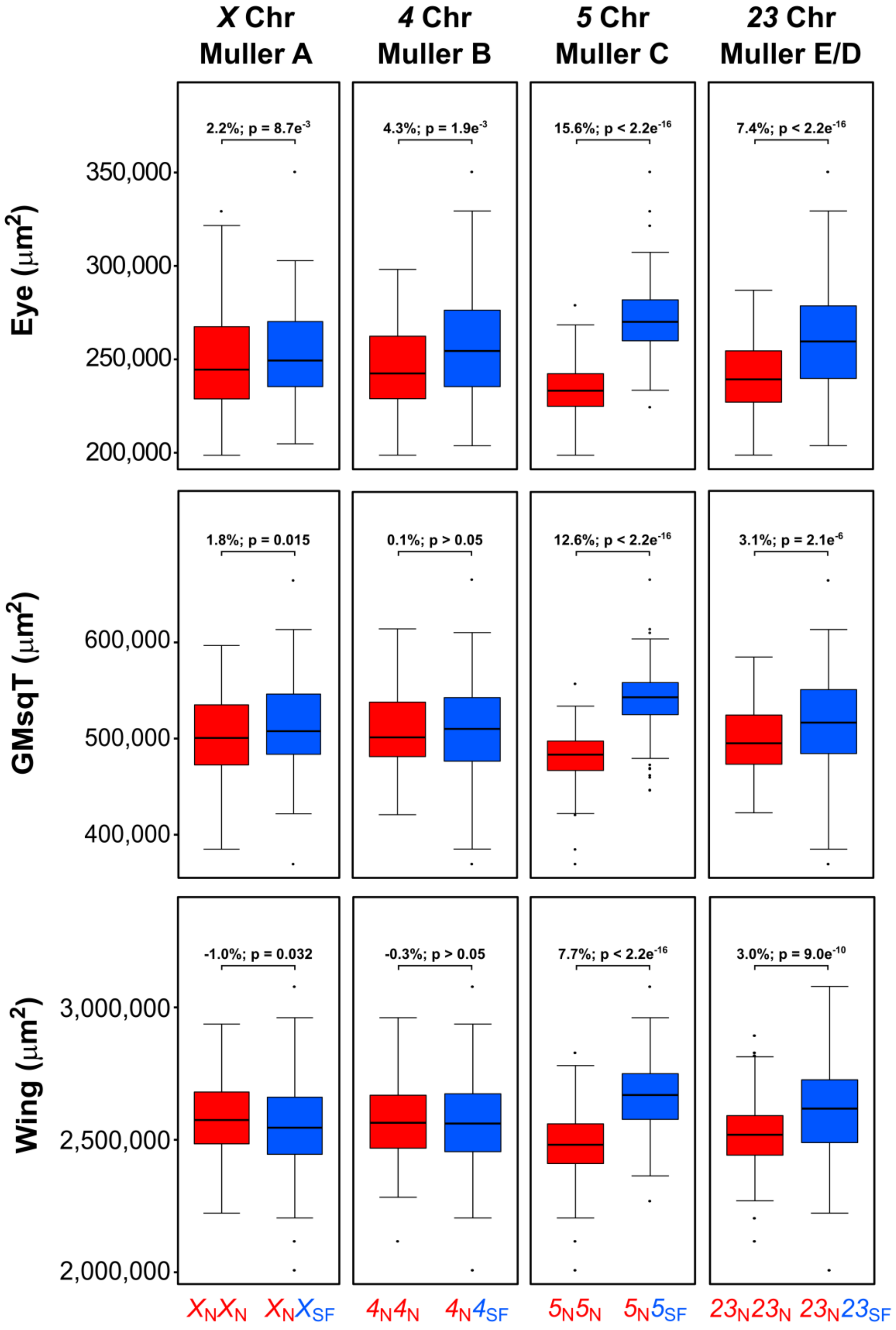

Supplementary Fig. S2. Variation in organ size in the genotype-phenotype associations using the backcross approach. Distributions of eye area, tibiae size (geometric mean of squared tibiae lengths (GMsqT)), and wing area for individuals homozygous for a given *D. novamexicana* chromosome and heterozygous *D. novamexicana*/*D. americana* for the respective chromosome. For each comparison, the percentage of difference and significance values after Wilcoxon-rank test are provided.

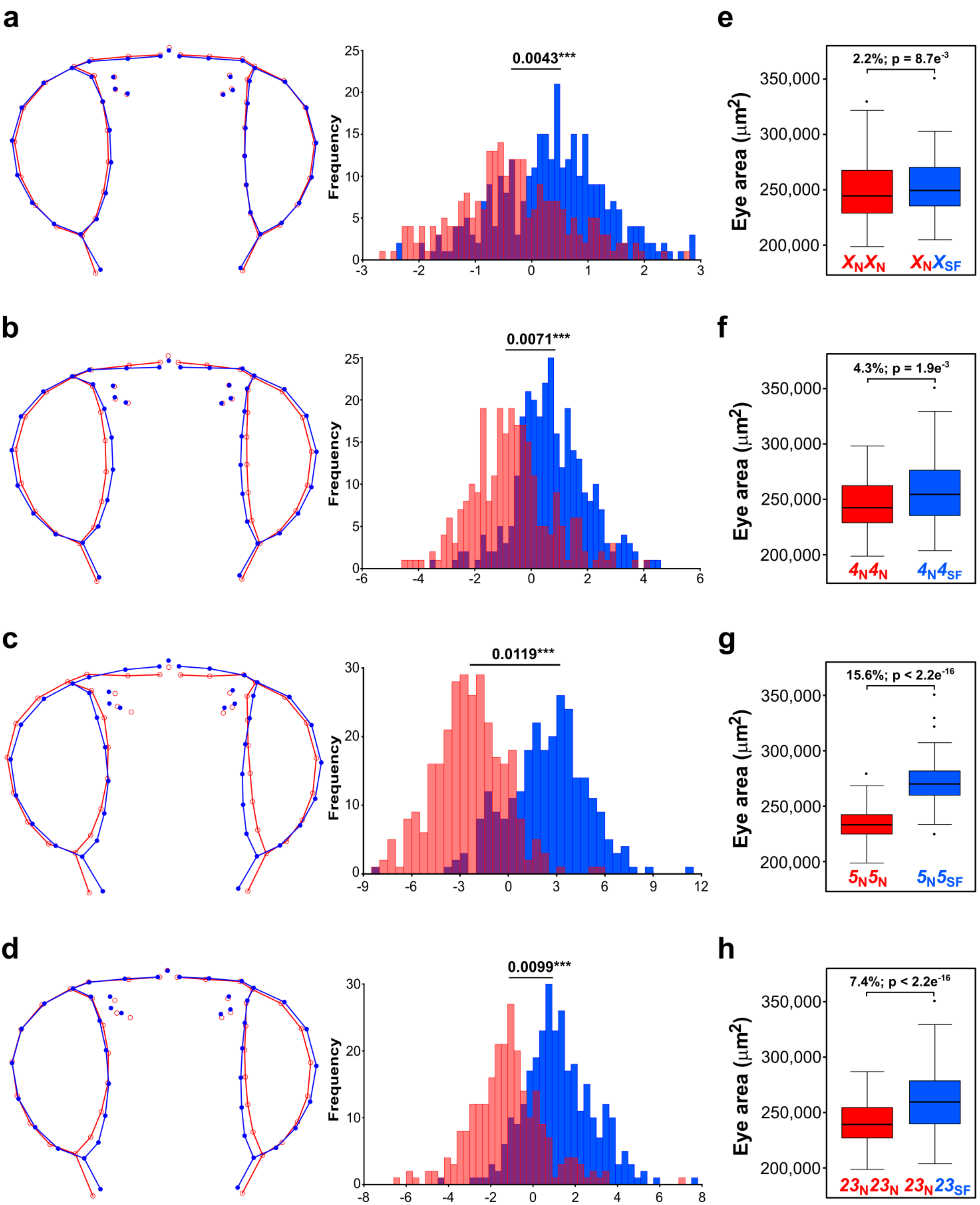
a
b
c
d
e
f
g
h
Frequency
0.0043***
0.0071***
0.0119***
0.0099***
Eye area (μm^2)
2.2%; p = 8.7e^{-3}
4.3%; p = 1.9e^{-3}
15.6%; p < 2.2e^{-16}
7.4%; p < 2.2e^{-16}
350,000
300,000
250,000
200,000
$X_N X_N$
$X_N X_{SF}$
$4_N 4_N$
$4_N 4_{SF}$
$5_N 5_N$
$5_N 5_{SF}$
$23_N 23_N$
$23_N 23_{SF}$

Supplementary Fig. S3. Variation in eye size and head shape in the genotype-phenotype associations using the backcross approach. a-d. Variation in mean head shape among the female progeny of the backcross between F1 hybrid females and *D. novamexicana* males. The histograms depict the frequency (*y*-axis) of the discriminant scores (*x*-axis) obtained after Discriminant Function Analysis (DFA) of the procrustes coordinates obtained from the first 19 principal components (90.8% of the total variation). Procrustes distances between groups are provided along with the histograms (*** = P < 0.0001 after a permutation test with 1,000 iterations). The wireframes depict changes in the mean shape multiplied by a factor of 5 (homozygous *D. novamexicana* (red) or heterozygous *D. novamexicana*/*D. americana* (blue)). **e-h** Distributions of eye size for females, progeny of the backcross, which were homozygous for a given *D. novamexicana* chromosome (red) or heterozygous *D. novamexicana*/*D. americana* (blue) for the respective chromosome. Information about the magnitude of change in eye size and the significance values is shown inside the graphs.

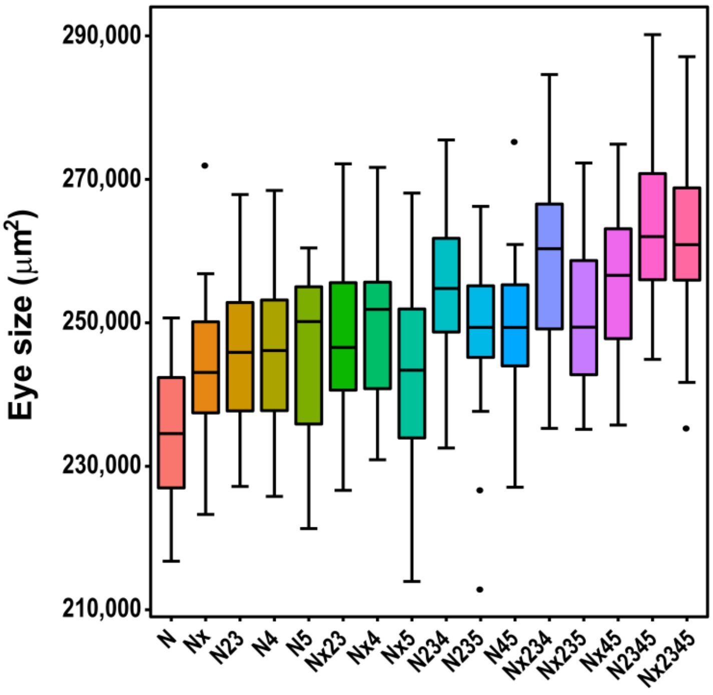

Supplementary Fig. S4. Relative eye size variation for the 16 genotypic classes present in the progeny of backcross between F1 hybrid females and *D. novamexicana* males. The residuals of the linear regression between eye area and tibiae lengths and wing areas were used to account for variation in body size. The grand mean of eye area was summed to the residuals to get relative eye size.

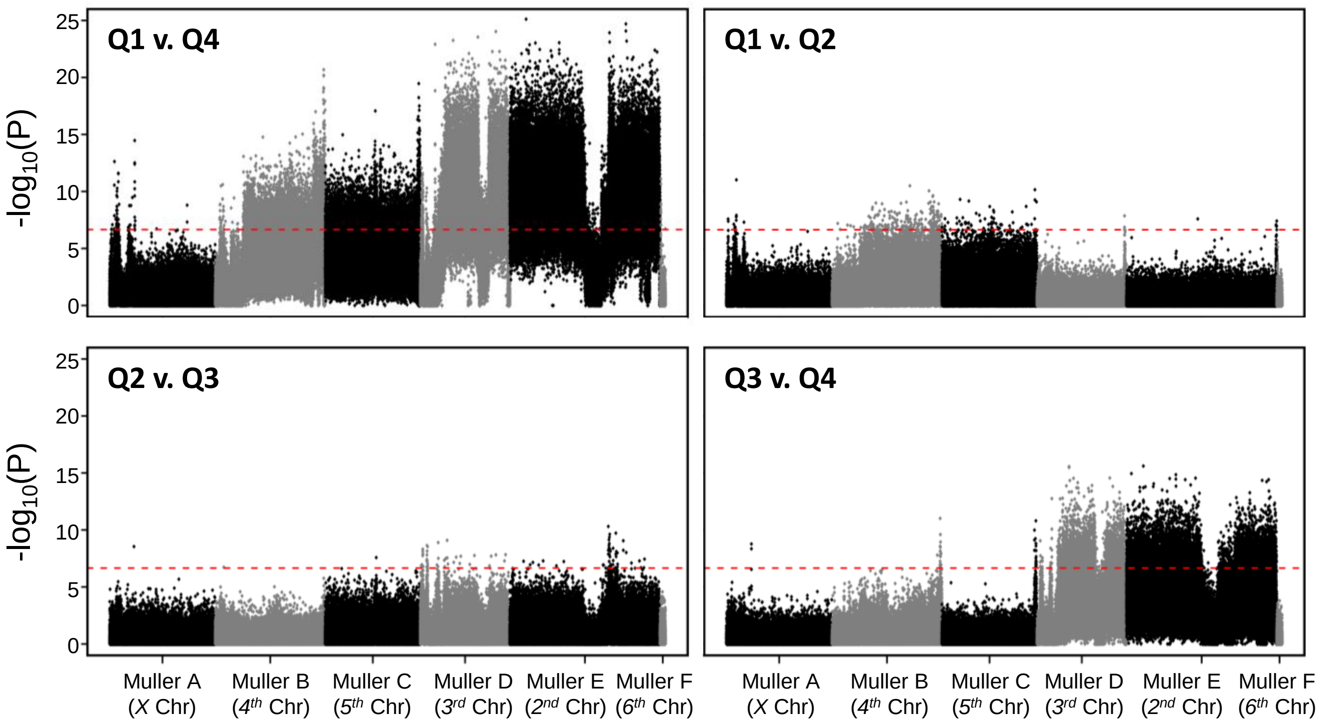

Supplementary Fig. S5. Manhattan plots between adjacent quartiles of the pool-seq experiment involving F18 females. The - $\log_{10}(P)$ values obtained after Fisher exact test were plotted in function of the location in the genome. The chromosomes are shown on the *x* axis and they are oriented always from telomere to centromere. The red lines represent the significant threshold after Bonferroni correction for multiple testing.

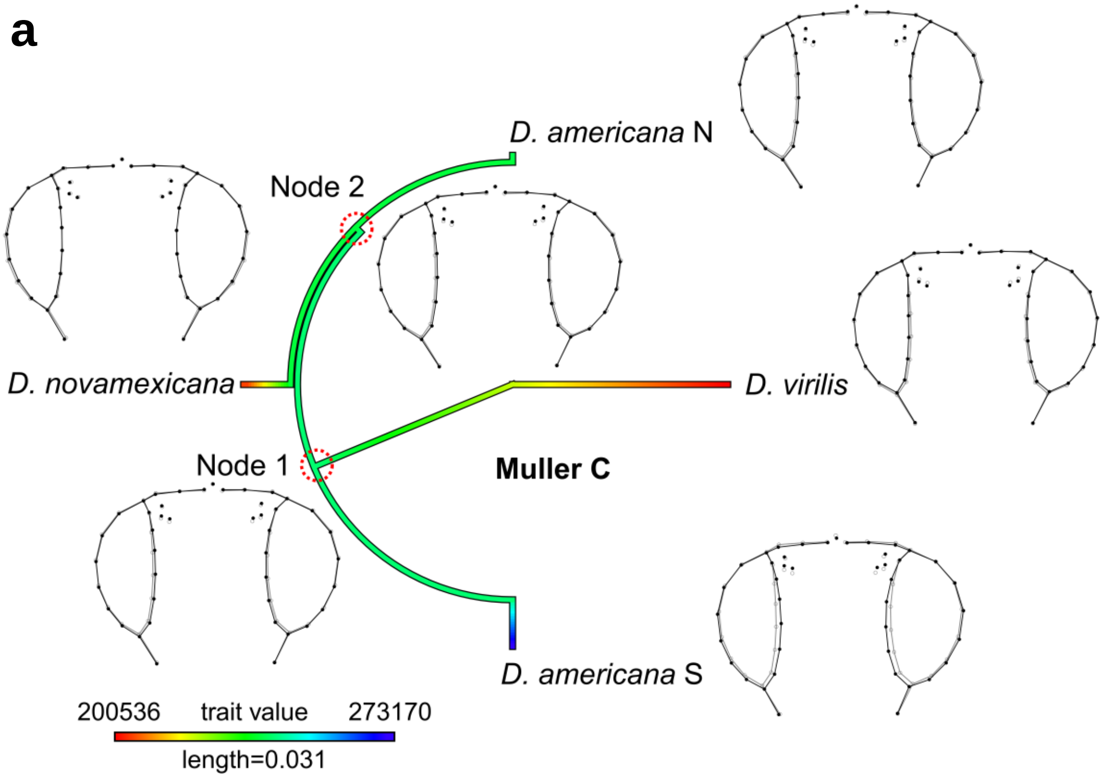

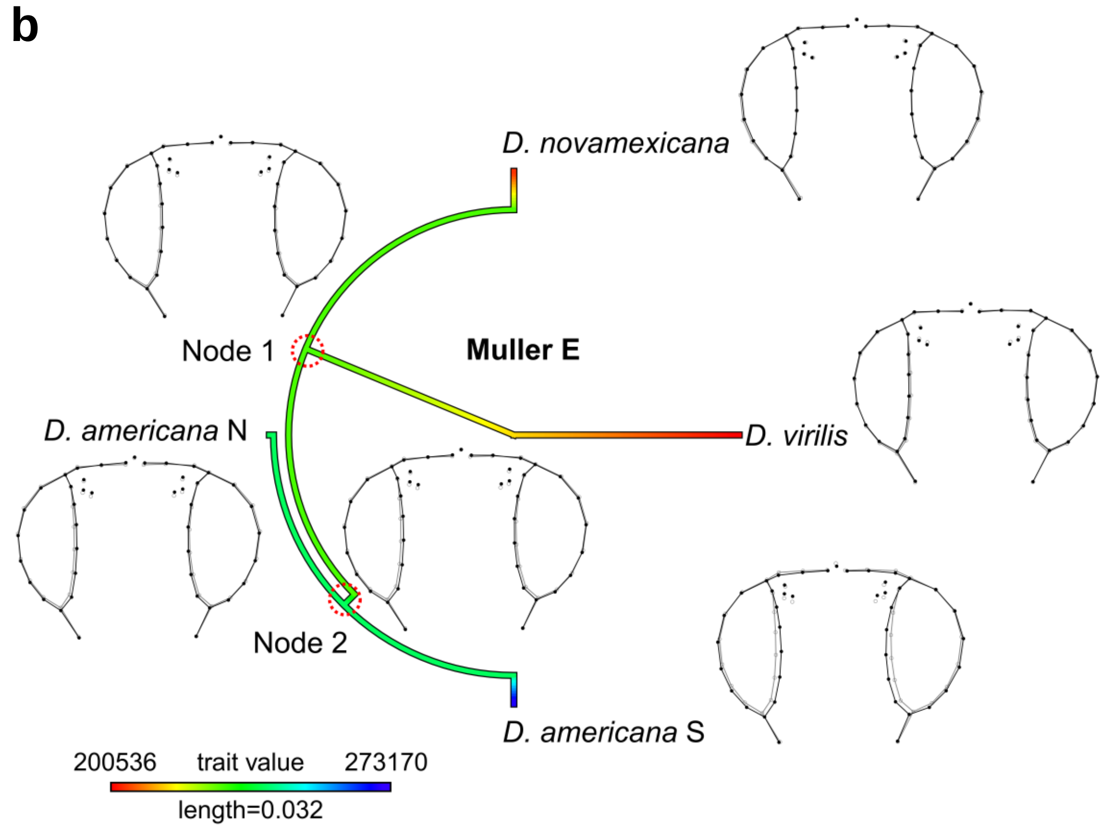

Supplementary Fig. S6. Phylogeny and ancestral reconstruction of the strains used in this study. a. Phylogeny based on genes located on the *5th* chromosome (Muller C). **b.** Phylogeny based on genes located on the *2nd* chromosome (Muller E). The wireframes (black – mean head shape of each species/population, grey – mean head shape of the estimated ancestral) are shown for each species/population.

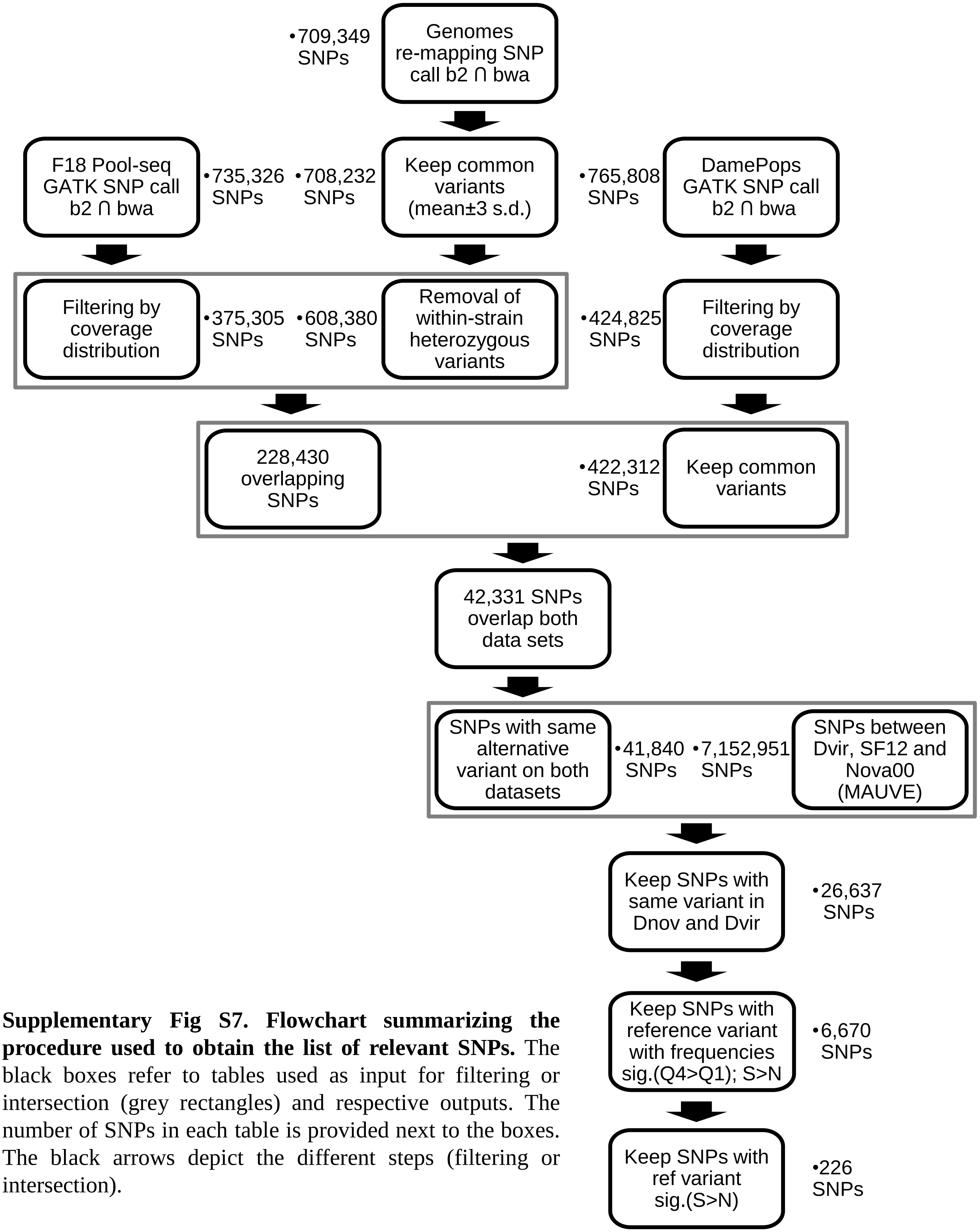

Supplementary Fig S7. Flowchart summarizing the procedure used to obtain the list of relevant SNPs. The black boxes refer to tables used as input for filtering or intersection (grey rectangles) and respective outputs. The number of SNPs in each table is provided next to the boxes. The black arrows depict the different steps (filtering or intersection).

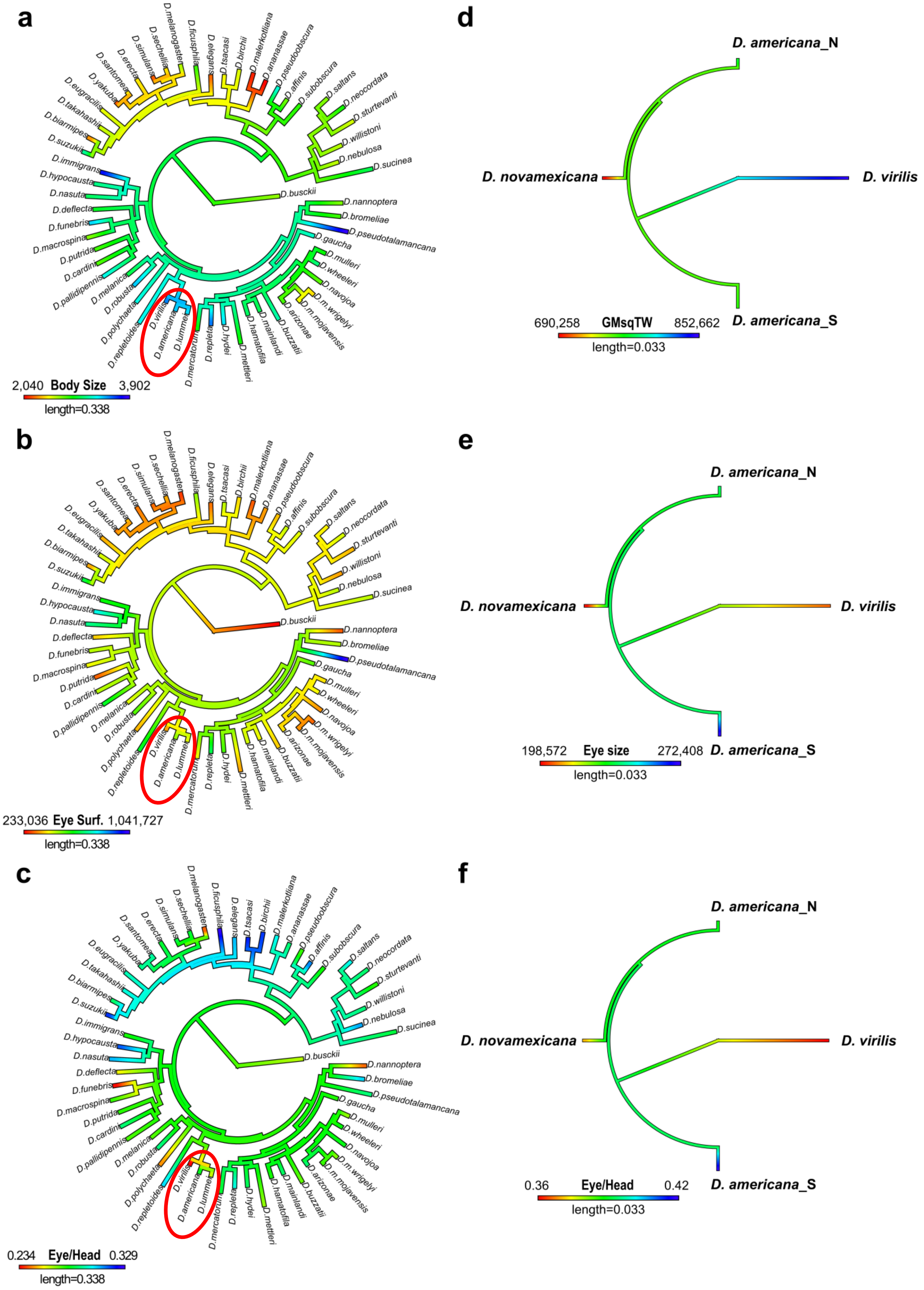
a
D.melanogaster
D.sechellia
D.simulans
D.erecta
D.santomea
D.yakuba
D.eugracilis
D.takahashii
D.biarmipes
D.suzukii
D.ficusphila
D.elegans
D.tsacasi
D.birchii
D.malerkotliana
D.ananassae
D.pseudoobscura
D.affinis
D.subobscura
D.saltans
D.neocordata
D.sturtevanti
D.willistoni
D.nebulosa
D.sucinea
D.busckii
D.immigrans
D.hypocausta
D.nasuta
D.deflecta
D.funebris
D.macrospina
D.putrida
D.cardini
D.pallidipennis
D.melanica
D.robusta
D.polychaeta
D.repletoides
D.virilis
D.americana
D.lummei
D.mercatorum
D.repleta
D.hydei
D.mettleri
D.hamatofila
D.mainlandi
D.buzzatii
D.arizonae
D.m.mojavensis
D.m.wrigelyi
D.navojoa
D.wheeleri
D.mulleri
D.gaucha
D.pseudotalamancana
D.bromeliae
D.nannoptera
2,040 Body Size 3,902
length=0.338
b
233,036 Eye Surf. 1,041,727
length=0.338
c
0.234 Eye/Head 0.329
length=0.338
d
D. americana_N
D. novamexicana
D. virilis
D. americana_S
690,258 GMsqTW 852,662
length=0.033
e
D. americana_N
D. novamexicana
D. virilis
D. americana_S
198,572 Eye size 272,408
length=0.033
f
D. americana_N
D. novamexicana
D. virilis
D. americana_S
0.36 Eye/Head 0.42
length=0.033

Supplementary Fig. S8. Comparison between the ancestral reconstruction of phenotypic traits across the *Drosophila* genus and the strains used in this study. a-c. The phylogeny and phenotypic data for body size (length from thorax to abdomen) (**a**), eye surface calculated from eye height and width (**b**) as well as eye length to head length ratios (**c**) were obtained from Keesey et al. (2019). The red ellipses depict species of the *virilis* group (*D. virilis, D. lummei* and *D. americana*). **d-f**. The phylogeny of the *virilis* group based on nucleotide sequences of genes located on the *4th* chromosome (Muller B). The phenotypes used for ancestral reconstruction were the following: geometric mean of tibia lengths and wing areas (proxy to body size) (**d**), eye area measured as the sum of the area of both eyes (**e**), and the ratios between eye area and head area (**f**).

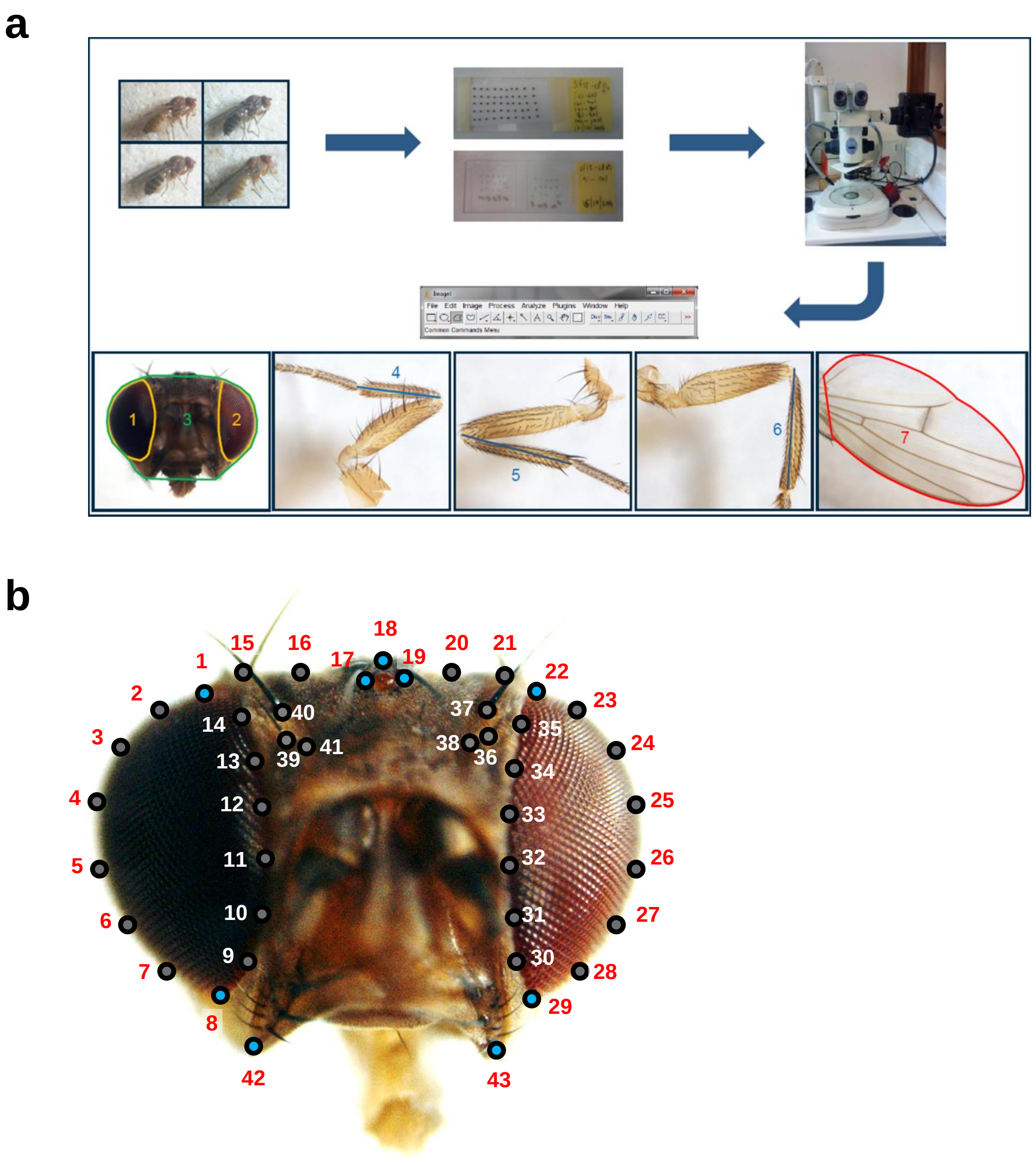

Supplementary Fig. S9. Schematic representation of the procedure used for phenotyping. a. Fly heads were dissected and mounted on sticky tape facing upwards. Tibiae and wings were mounted in Hoyer's medium after dissection. Pictures were taken using a camera attached to a stereomicroscope. Eye area was determined by the area defined by the outlines 1 and 2 while the face cuticle was determined by subtracting eye area to complete head area [3-(1+2)]. Tibiae lengths were measured as the distances represented by 4, 5, and 6 for tibia 1, 2 and 3, respectively. Wing area was calculated by measuring the area defined by the outline represented by 7. **b.** A total of 11 fixed landmarks (blue) and 32 semi-landmarks (grey) were placed on frontal pictures of heads for geometric morphometrics analysis (see Material and Methods for details).

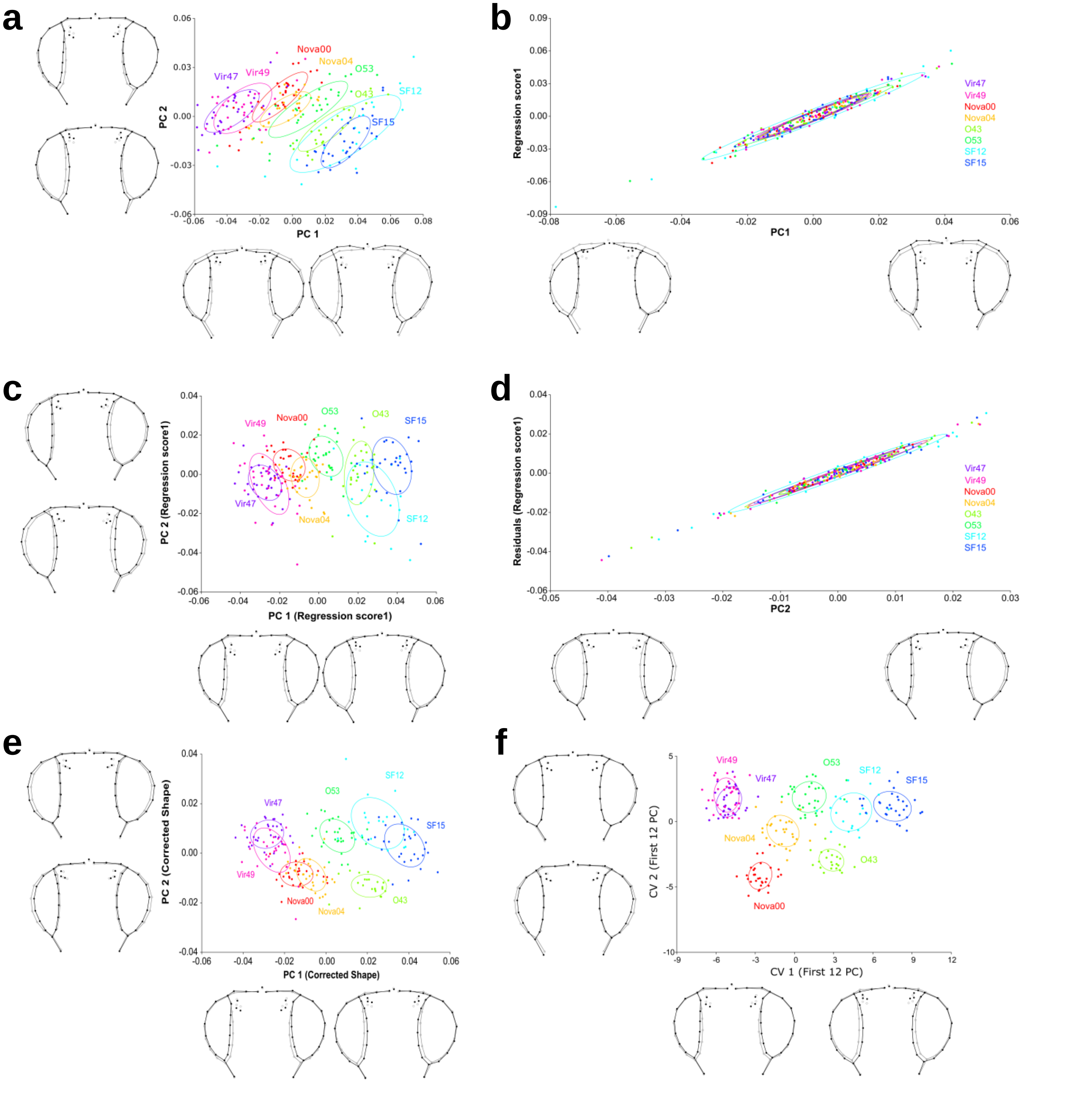

Supplementary Fig. S10. Sequential removal of error associated with head tilting. a. Principal Component Analysis (PCA) of shape of species of the *virilis* group. **b.** Strain-centred regression of shape on PC1 capturing variation associated with roll (up/down). **c.** PCA of the residual variation of shape on PC1 (shape corrected for roll). **d.** Strain-centred regression of shape corrected for roll on PC2 capturing variation associated with yaw (left/right). **e.** PCA of the residual variation of shape corrected for roll on PC2 (shape corrected for yaw). **f.** Canonical Variate Analysis of shape corrected for roll and yaw. The wireframes depict changes in shape along the two main axes of variation (black - the maximum and minimum values on the axis (Mahalanobis distances); grey – mean shape for each axis). The equal frequency ellipses are given with probability of 0.5.

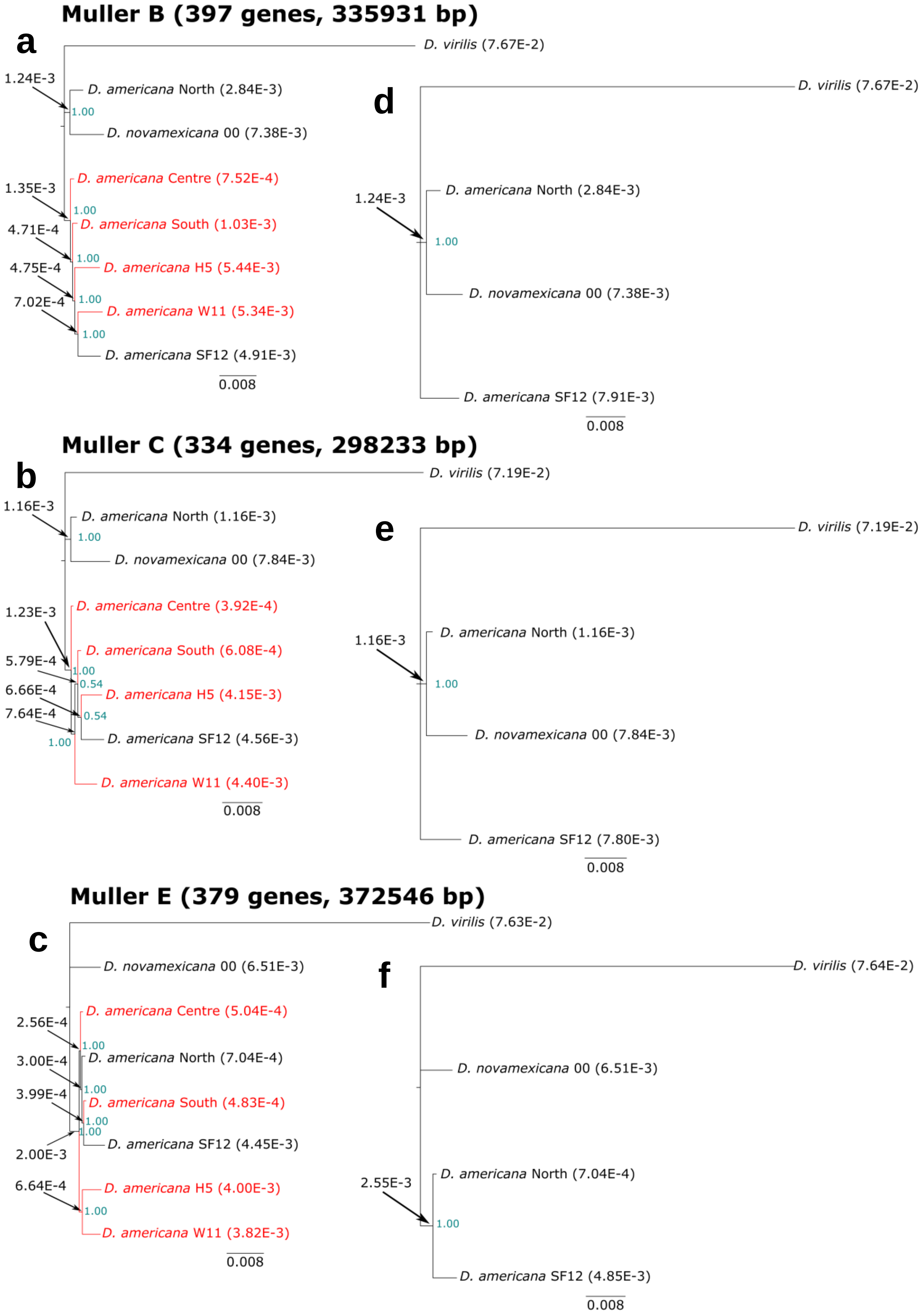

Supplementary Fig. S11. Phylogenies of species of the *virilis* phylad. a-c. Unrooted phylogenies based on genes on the *4th*, (a) *5th*, (b), and *2nd* (c) chromosomes (Muller elements B, C, and E). **d-f.** Edited phylogenies to include only the strains/populations used in this study for the *4th*, (d) *5th*, (e), and *2nd* (f) chromosomes.

List of additional supplementary files online (provided as Excel files)

Supplementary File S1. Descriptive statistics for all datasets.

Supplementary File S2. List of primers used as molecular markers for chromosomal inversions and genotyping results.

Supplementary File S3. SNP tables after intersecting the datasets obtained for the GWAS and *D. americana* populations and candidate genes for eye development.

Supplementary File S4. Raw measurements.

Supplementary File S5. List of primers used as indel markers for the different chromosomes and genotyping of the progeny of the backcross between hybrid females and *D. novamexicana* males.
